# EGR1 Mediates Ursodeoxycholic Acid‐Promoted Mitophagy to Prevent Postovulatory Aging of Porcine Oocytes

**DOI:** 10.1111/acel.70612

**Published:** 2026-07-03

**Authors:** Ying Zhang, Qianru Han, Yongchao Liu, Wei Shen, Shunfeng Cheng, Xiaoya Li

**Affiliations:** ^1^ College of Animal Science and Technology, Shandong Engineering Research Center for Protection of Livestock and Poultry Genetic Resources and Biological Breeding Qingdao Agricultural University Qingdao China; ^2^ College of Life Sciences, Qingdao Agricultural University Qingdao China

**Keywords:** early embryonic development, EGR1, mitophagy, porcine, postovulatory oocyte aging, ursodeoxycholic acid

## Abstract

Postovulatory oocyte aging (POA) is a key factor contributing to the decline in female fertility and the success rate of assisted reproductive technology. Currently, most studies on POA have focused on downstream phenotypes such as mitochondrial dysfunction and oxidative stress, while little is known about its key upstream regulatory factors. Here, we show that the downregulation of transcription factor Early Growth Response 1 (EGR1) is a key upstream event driving porcine oocyte aging. Microtranscriptome sequencing combined with experimental validation verified a notable reduction in EGR1 protein abundance in aged oocytes. We found that Ursodeoxycholic Acid (UDCA) upregulated EGR1, which in turn promoted the expression of the autophagy‐related protein LC3B and the lysosomal protein LAMP1, while reducing P62 accumulation. Furthermore, UDCA enhanced the expression of mitophagy core proteins PINK1, VDAC1 and promoted mitochondrial‐lysosomal colocalization, thereby improving mitophagy and restoring the quality of aged oocytes. Crucially, treatment with the EGR1 inhibitor plicamycin completely blocked UDCA's ability to enhance the developmental potential of aged oocytes, confirming that EGR1‐mediated mitophagy was the core pathway underlying UDCA's effects. Collectively, this study innovatively identified EGR1 as a key bridge linking oocyte aging and decreased mitophagy, and clarified the novel mechanism by which UDCA exerts its protective effects through the “UDCA‐EGR1‐mitophagy” axis. Our findings advanced the research on oocyte aging from phenotypic observation to the upstream transcriptional regulation level, providing a novel theoretical target and experimental basis for fundamentally intervening in reproductive aging.

## Graphical abstract

This study delivers dual key advances: mechanistically, we uncover a novel role of EGR1 as a pivotal transcriptional regulator that bridges upstream gene expression dysregulation to downstream mitophagic decline in oocyte aging; and translationally, we validate the therapeutic potential of the clinical drug UDCA to counteract oocyte aging.

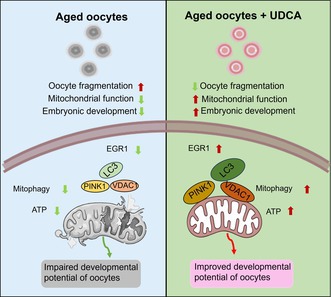

## 1 Introduction

Ovulated oocytes that fail to be fertilized within the optimal timeframe undergo a time‐dependent decline in quality, known as postovulatory aging (POA) (Ma et al. 2025) POA is a key factor contributing to the decline in female fertility (Miao et al. 2020). POA is always accompanied by a series of adverse cellular changes, including elevated oocyte fragmentation, abnormal chromosome alignment, spindle assembly defect, partial exocytosis of cortical granules (CGs), oxidative stress and apoptosis (Park et al. 2025). One of the most prominent phenotypes of oocyte aging is the decline in mitochondrial function, characterized by a marked reduction in mitochondrial membrane potential (MMP), normal mitochondrial distribution rate and ATP production capacity (Liu et al. 2023). Studies have shown that aged oocytes impaired the uniform distribution of mitochondria, leading to massive aggregation and a consequent reduction in the developmental potential of porcine oocytes (Luo et al. 2020). Additionally, aged oocytes compromised the quality of porcine oocytes by decreasing JC‐1 and ATP levels in mitochondria (Nie et al. 2019). Consequently, mitochondrial dysfunction undermines oocyte quality, which ultimately impairs fertilization potential and reduces the developmental competence of the embryo (Zhang, Keilty, et al. 2017; Zhang, Tong, et al. 2017). Over the past few years, a multitude of studies have demonstrated that the primary cause of quality deterioration in aged oocytes was the reduction in mitophagy levels (Cox et al. 2021). Previously, research has reported that in oocytes from aged mice, the autophagy‐related protein LC3B exhibited significantly decreased expression levels, while the protein level of P62 was abnormally elevated, indicating a decline in autophagic activity, which ultimately impaired oocyte quality (Liang et al. 2020). Furthermore, the downregulation of mitophagy marker PINK1 and the outer mitochondrial membrane protein VDAC1 in oocytes from aged mice suppressed in vitro maturation (IVM) activity, leading to a decline in oocyte quality (Zhang et al. 2025, 2023). In addition, lysosomal function and the expression level of lysosomal membrane protein LAMP1 were significantly decreased in aged oocytes, impairing lysosome‐mediated autophagy and thereby compromising oocyte quality (Zhou et al. 2019). Collectively, the present study's outcomes reveal that reduced mitophagy acts as a crucial driver of oocyte aging (Zhang et al. 2023). Currently, research on oocyte aging primarily focuses on phenotypes, such as mitophagy, mitochondrial function, apoptosis and oxidative stress (Bai et al. 2024; Ma et al. 2025; Zhang et al. 2025, 2022, 2024), whereas studies on the aging mechanism of porcine oocytes are rarely reported.

One key feature of postovulatory oocyte aging is mitochondrial dysfunction and impaired autophagic/mitophagic flux, a critical upstream event for oocyte quality decline (Zhang et al. 2023). Therefore, targeted enhancement of the autophagy pathway is a potential strategy for rescuing the function of aging oocytes. Ursodeoxycholic acid (UDCA), a clinically safe bile acid drug, which has been widely used in clinical practice and extensively studied for its pharmacological activities. Yan et al. (2009) established an HPLC‐MS/MS method to determine UDCA levels in human plasma and detected related drug concentrations in 20 healthy volunteers after a single oral administration of 500 mg UDCA under fasting conditions, with key pharmacokinetic parameters documented (Yan et al. 2009). The peak plasma UDCA concentration was 7.18 ± 2.40 μg/mL. After conversion based on the molecular weight of UDCA (392.58 g/mol), this value equals 18.3 ± 6.1 μM. The reported analytical method exhibited high sensitivity, accuracy and specificity for human UDCA pharmacokinetic analysis, providing reliable plasma concentration references for both clinical practice and basic research. In addition, Sathe et al. (2020) reported that UDCA at a plasma concentration of approximately 22 μM exerted neuroprotective effects by enhancing brain mitochondrial energy metabolism and elevating ATP levels, thereby maintaining neuronal function in patients with Parkinson's disease (Sathe et al. 2020). These studies indicate that the peak plasma concentration of UDCA in humans falls in the range of 15–30 μM following oral administration of conventional clinical doses. Furthermore, UDCA has demonstrated robust cytoprotective effects in multiple pathological models, with its core mechanism tightly linked to autophagy and mitochondrial homeostasis regulation (Bell et al. 2018; Chen et al. 2021; Mortiboys et al. 2015). Studies indicated that UDCA mitigated non‐alcoholic fatty liver disease by triggering the activation of AMPK and subsequent enhancement of autophagy (Wu et al. 2020). Furthermore, UDCA activated the PINK1/Parkin pathway, thereby restoring autophagic flux and promoting mitophagy, which ultimately led to improved behavioral deficits in Parkinson's disease mice (Payne et al. 2023). Moreover, UDCA suppressed the proliferation of hepatocellular carcinoma by inducing autophagy through upregulation of LC3B expression (Wang et al. 2017). Importantly, UDCA alleviated Alzheimer's disease symptoms by enhancing mitochondrial membrane potential through the regulation of Drp1 levels and localization (Bell et al. 2018). Additionally, research in animal models has confirmed that UDCA alleviated the pathology induced by the Parkinson's disease‐associated LRRK2^G2019S^ mutation by increasing ATP levels (Mortiboys et al. 2015). Notably, while these effects of UDCA have been primarily characterized in somatic cells and disease models, the core mechanism of autophagy‐mitochondrial network regulation is evolutionarily conserved, implying its potential applicability in germ cells such as oocytes. Based on this, we hypothesize that UDCA may reverse functional deterioration during postovulatory oocyte aging by activating the mitophagic pathway and restoring mitochondrial homeostasis. This study aims to verify and elucidate the novel mechanism underlying UDCA‐mediated regulation of reproductive aging using the porcine oocyte aging model.

Early growth response protein 1 (EGR1), alternatively named NEFI‐A, ZIF268, KROX‐24 as well as TIS85, belongs to the EGR family and serves as a pivotal transcription factor (Wang et al. 2021). EGR1 primarily regulates critical physiological processes including apoptosis, proliferation, and differentiation (Chen et al. 2022; Chiba et al. 2022; Lan et al. 2023). Research has demonstrated that in the central nervous system of mice, EGR1 inhibited neuronal apoptosis by activating the TGFβ1 pathway and reducing the accumulation of mitochondrial ROS (Lan et al. 2023). Moreover, EGR1 bound to the SOX9 promoter, activating the Wnt/β‐catenin pathway, which promoted the proliferation of renal tubular cells and thereby accelerated kidney repair (Chen et al. 2022). Furthermore, EGR1 enhanced bovine skeletal muscle satellite cell differentiation by elevating MyoG gene expression (Zhang, Keilty, et al. 2017; Zhang, Tong, et al. 2017). In addition, EGR1 enhanced the phosphorylation of SMAD1/5, thereby enhancing the differentiation efficiency of osteoblasts (Chiba et al. 2022). Recent studies have reported that EGR1 was implicated in the regulation of mitophagy and the aging process (Chen et al. 2008; Krones‐Herzig et al. 2003). EGR1 upregulated the expression of LC3 by directly binding to its promoter, enhanced autophagy, and ultimately led to the deterioration of chemotherapy efficacy in hepatocellular carcinoma cells (Peng et al. 2016). Conversely, EGR1 exacerbated intervertebral disc degeneration in nucleus pulposus cells by inhibiting the Pink1‐Parkin pathway of mitophagy (Wu et al. 2024). In parallel, numerous studies have revealed that EGR1 impeded the senescence of colon cancer cells by binding to the SIRT6 promoter and inhibiting its protein expression (Xu et al. 2024). On the other hand, some studies report that EGR1 activated the p53/p21 signaling pathway to promote senescence in lung cancer cells, thereby alleviating the malignant progression of the disease (Krones‐Herzig et al. 2003). In conclusion, while EGR1 demonstrates clearly context‐dependent functions with distinct effects in various cell lineages, its specific role and mechanism in aging porcine oocytes remain largely unexplored.

Therefore, this study established an aging model using porcine oocytes cultured in vitro and employed transcriptomics to investigate the impacts of UDCA on postovulatory aged porcine oocytes. We found that aging inhibited mitophagy by downregulating EGR1, inducing mitochondrial dysfunction, oxidative stress, and apoptosis. Furthermore, aging reduced the developmental potential of early embryos. EGR1 upregulation‐mediated mitophagy promotion by UDCA contributed to the restoration of quality in aging‐impaired porcine oocytes. Our findings provided new insights into the molecular regulatory mechanisms underlying porcine oocyte aging.

## 2 Materials and Methods

### 2.1 Ethics Statement

In the present study, all porcine ovarian tissue samples herein were sourced as byproducts from a government‐registered commercial slaughterhouse. Meanwhile, all study protocols were reviewed and approved by the Laboratory Animal Management and Use Committee of Qingdao Agricultural University (Approval Number: 2021–0017).

### 2.2 Experimental Design

Cumulus‐oocyte complexes (COCs) with normal morphology and intact cumulus cell layers were collected from porcine ovaries and cultured in vitro maturation (IVM) medium for 44 h at 38.5°C under 5% CO_2_ to complete oocyte maturation. Mature COCs without prolonged additional culture were directly used for subsequent experiments and defined as the Fresh group. Another batch of mature COCs with intact cumulus cells was transferred to fresh IVM medium and cultured for an additional 48 h to establish the Aged group. The Aged + UDCA group was treated identically to the Aged group, except that UDCA was added to the fresh IVM medium during medium replacement. These COCs were further cultured for 48 h under the same conditions to simulate POA. Subsequently, the oocytes were randomly assigned to additional treatment groups: Fresh, Aged, Aged + 5 UDCA (Aged exposure with 5 μM UDCA), Aged + 10 UDCA (Aged exposure with 10 μM UDCA), Aged + 15 UDCA (Aged exposure with 15 μM UDCA), Aged + 25 UDCA (Aged exposure with 25 μM UDCA), Aged + 50 UDCA (Aged exposure with 50 μM UDCA), Aged + 75 UDCA (Aged exposure with 75 μM UDCA) and Aged + 100 UDCA (Aged exposure with 100 μM UDCA).

### 2.3 Antibodies

EGR1 (Cat#: 15F7, Cell Signaling Technology), LC3B (Cat#: ab51520, Abcam), P62 (Cat#: A19700, Abclonal), LAMP1 (Cat#: C54H11, Cell Signaling Technology), VDAC1 (Cat#: A19707, Abclonal), PINK1 (Cat#: DF7742, Affinity).

### 2.4 Chemical

Ursodeoxycholic acid (UDCA) (Cat#: U5127, Sigma‐Aldrich) was solubilized in dimethyl sulfoxide (DMSO) and subsequently stored at −20°C. The concentration of UDCA stock solution was 100 mM (Chun and Low 2012; Jung and Hwang 2021). Preceding application, UDCA was diluted in culture medium to achieve the target final concentration, with the highest concentration of UDCA employed in the experiment being 100 μM. Notably, the final DMSO concentration in the oocyte culture medium was 0.1%, which proved non‐interfering with the cell‐based assay. Meanwhile, to ensure experimental accuracy and rigor, consistent DMSO concentrations were maintained across all treatment groups; Plicamycin (MMA) (Cat#: HY‐A0122, MCE) was dissolved in DMSO and stored at −20°C. The concentration of MMA stock solution was 1 mM. Before use, the final concentration of MMA was diluted with culture medium. MMA was used at a maximum concentration of 200 nM (Choi et al. 2014; Saranaruk et al. 2020; Shen, Lu, et al. 2022; Shen, Wu, et al. 2022), and final DMSO concentration in oocyte culture medium was 0.2%, exerting no interference on cell experiments. Meanwhile, to ensure experimental accuracy and rigor, across all treatment groups, DMSO concentrations were maintained uniform.

### 2.5 Preparation of PZM‐3 Embryo Culture Medium

To 100 mL of ultrapure water, the following components were added sequentially: 0.6312 g NaCl, 0.0746 g KCl, 0.0048 g KH_2_PO₄, 0.0099 g MgSO₄·7H_2_O, 0.2106 g NaHCO₃, 0.0146 g L‐glutamine, 0.0022 g sodium pyruvate, 0.0617 g calcium lactate, 0.0546 g taurine, and 0.0050 g gentamicin. After stirring until completely dissolved, 2.0 mL β‐mercaptoethanol, 1.0 mL non‐essential amino acid solution, and 0.3 g BSA were added. The solution was thoroughly mixed, filter‐sterilized using a 0.22 μm filter, and stored at 4°C. All reagents were obtained from Sigma‐Aldrich unless otherwise specified.

### 2.6 In Vitro Culture of Porcine Oocytes

Porcine ovaries were harvested from a local commercial abattoir (Wanfu Group, Qingdao, China) and further carried to the laboratory within 2 h in an insulated flask maintained at a constant temperature of 37°C, which was supplemented with 3% penicillin–streptomycin solution. Follicular fluid was aspirated from 5 to 6 mm diameter follicles using a single‐use 10‐ml disposable syringe. COCs exhibiting uniform cytoplasm and surrounded by 3–5 layers of granulosa cells were screened. Subsequently, the samples were subjected to washing with TL‐HEPES operating medium supplemented with 1% antibiotic mixture, 10% serum, and 2% HEPES stock solution. Subsequently, oocytes were transferred to IVM medium. IVM medium included 0.5 ng/mL fibroblast growth factor, 3.05 mM glucose, 0.57 mM cysteine, 0.91 mM sodium pyruvate, 2.5 ng/mL epidermal growth factor, 10% polyvinyl alcohol, 2.5 ng/mL recombinant porcine interleukin‐6, 100 IU/mL pregnant mare serum gonadotropin, 100 IU/mL human chorionic gonadotropin, 2.5 ng/mL keratinocyte growth factor, 250 IU/mL follicle‐stimulating hormone, 1% antibiotics and the remainder was MI99. All reagents were obtained from Sigma‐Aldrich unless otherwise specified.

### 2.7 In Vitro Culture of Porcine Blastocysts

After 44 h of IVM, oocytes with the first polar body expelled were selected to undergo parthenogenetic activation. Selected oocytes were subjected to parthenogenetic activation with 10 μM calcium ionomycin (Cat#13909, Sigma) under dark conditions, followed by three washes in 50 μL culture droplets, and subsequent incubation in a 38.5°C incubator supplemented with 5% CO_2_ for 5 min. Afterwards, the oocytes were washed three times in 50 μL droplets containing 6‐Dimethylaminopurine (Cat# D2629, Sigma) and incubated in an incubator for 5 h. Oocytes were then transferred to pre‐equilibrated 4‐well plates containing embryo culture medium (PZM‐3) and cultured in the incubator. The embryos were observed at 24 h for the 2‐cell stage, at 48 h for the 4‐cell stage and at 168 h for the blastocyst stage.

### 2.8 Detection of ROS

Oocytes were incubated in PBS supplemented with 10 μM DCFH‐DA (Beyotime Biotechnology, China) for a duration of 30 min. Subsequently, the oocytes were moved to 0.1% PVA‐PBS buffer and subjected to washing. Oocytes were immediately observed and imaged via a confocal microscope (Agilent Leica TCSspII, USA), and the fluorescence signals of oocytes in each group were subjected to quantitative analysis. Meanwhile, the fluorescence intensity of oocytes in each experimental group was analyzed using Image J software. Independent experiments were repeated five times to verify the reliability of results (*n* = 120–150 COCs).

### 2.9 Detection of Mitochondrial Distribution

Oocytes were placed into MitoTracker Red CMXRos solution (Meilunbio, China), which was diluted at a ratio of 1:1000 using blocking buffer, then maintained for 30 min in an incubator with 5% CO_2_ at 38.5°C. The oocytes were then subjected to washing treatment in 0.1% PVA‐PBS buffer, transferred to a glass slide with anti‐fluorescence quencher containing Hoechst 33342 and mounted with a coverslip whose four corners were coated with lanolin. Next, oocytes were immediately photographed and observed under a confocal microscope to examine the mitochondrial distribution in oocytes of each group, and the proportion of abnormal mitochondrial distribution was determined. Independent experiment was performed with a minimum of five replicates (*n* = 100–120 COCs).

### 2.10 Detection of Early Apoptosis

Oocytes were transferred to the pre‐prepared Annexin V‐FITC staining working solution (Beyotime Biotechnology, China), followed by incubation in an incubator with 5% CO_2_ at 38.5°C for 30 min. Subsequently, the oocytes were rinsed in 0.1% PVA‐PBS, with each wash lasting 5 min. Finally, oocytes were transferred into droplets, photographed under a confocal microscope to evaluate the apoptosis staining results of oocytes within each experimental group, and the apoptosis rate was calculated. Independent experiment was performed with a minimum of five replicates (*n* = 125–150 COCs).

### 2.11 Detection of MMP

The oocytes were first subjected to washing in PVA‐PBS buffer, and subsequently incubated with 10 μg/mL JC‐1 (Beyotime Biotechnology, China) for a 30 min duration in an incubator. Then, oocytes were transferred to PVA‐PBS culture droplets and visualized via a laser scanning confocal microscope. The fluorescence intensity of individual oocytes was evaluated under standardized scanning parameters. Relative fluorescence intensities in oocytes were measured with ImageJ software. The level of mitochondrial membrane potential was determined according to the red/green fluorescence pixel proportion. Independent experiment was performed with a minimum of five replicates (*n* = 100–110 COCs).

### 2.12 ATP Level Measurement

Intracellular ATP levels in oocytes were quantitatively measured using an ATP detection kit purchased from Beyotime Biotechnology (Cat# S0027, China), strictly following the manufacturer's standard protocol. Briefly, following in vitro culture, oocytes were transferred into microdrops of 0.1% PVA‐PBS prewarmed to 37°C and washed three times thoroughly to eliminate residual culture medium interference. Subsequently, samples were grouped according to the required sample size, with 50 oocytes pooled as one technical replicate and five biological replicates set for each group. The prepared samples were lysed thoroughly with lysis buffer according to the kit instructions, mixed with ATP detection working solution, and then measured for relative light units using a multimode microplate reader in chemiluminescence mode. To ensure the reliability and reproducibility of the experimental results, the entire experiment was independently repeated five times (*n* = 120–150 COCs).

### 2.13 CGs Staining

Oocytes were transferred to 0.1% PVA‐PBS and washed three times, then underwent fixation treatment in 4% PFA for 30 min, incubated in permeabilization solution for 20 min, subjected to blocking in 1% BSA for a duration of 1 h, and incubated in 100 μg/mL LCA‐FITC (Xi'an ruixi Biological Technology Co. Ltd) at room temperature under dark conditions for a duration of 30 min. After that, they were washed 5 times in 0.1% PVA‐PBS (5 min each time). Finally, they were transferred to a glass slide with anti‐fluorescence quencher containing Hoechst 33342, and the slide was covered with a coverslip whose four corners were coated with lanolin for mounting. Images were acquired via confocal microscopy, and the cortical granule fluorescence intensity was enumerated for each group. Independent experiments were repeated five times to verify the reliability of results (*n* = 100–110 COCs).

### 2.14 Lysosome Staining

Oocytes were cultured with 1 mM lysosomal dye (Beyotime Biotechnology, China) for 1 h at 38.5°C in a 5% CO_2_ incubator. After that, oocytes underwent five washes with 0.1% PVA‐PBS buffer, with each wash duration lasting 5 min. Finally, droplets were prepared and photographed under a confocal microscope, and the lysosomal fluorescence intensity of each group was subjected to quantitative measurement. Independent experiments were repeated five times to verify the reliability of the results (*n* = 120–130 COCs).

### 2.15 Immunofluorescence Staining

Oocytes were transferred to pre‐warmed 0.1% PVA‐PBS droplets and washed 3 times. Afterward, oocytes were fixed with 4% PFA (30 min), permeabilized with 0.5% Triton X‐100 in PBS (20 min), and blocked with 1.0% BSA (1 h); then, they were incubated with LC3B (1:200), LAMP1 (1:200), P62 (1:200), VDAC1 (1:200), PINK1 (1:200), and EGR1 (1:500) at 4°C overnight. After undergoing three rounds of PBS washing, the oocytes were exposed to goat anti‐rabbit IgG secondary antibody (Abcam) diluted 1:200 in blocking buffer, with a 1 h incubation at room temperature. Following 5 min staining using 10 μg/mL Hoechst 33342, oocytes were rinsed with PBS three times and mounted on prepared slides afterward. Independent experiments were repeated five times to verify the reliability of results (*n* = 100–120 COCs).

### 2.16 Detection of Spindle Organization and Chromosome Alignment

Oocytes were transferred to pre‐warmed 0.1% PVA‐PBS for washing. Oocytes underwent fixation in 4% PFA for 30 min, permeabilization in 0.5% Triton X‐100‐containing buffer for 20 min, and blocking with 1% BSA for 1 h. They were then stained with α‐tubulin conjugated to 488 fluorescence green (1:500) for 2 h at room temperature under light‐free conditions. After that, the specimens received five rounds of 5‐min washing with 0.1% PVA‐PBS, then subjected to nuclear staining with PI for 3 min, and subsequently washed three times with 0.1% PVA‐PBS. Finally, oocytes were placed on coverslips for mounting and subsequently imaged with a confocal microscope to examine the morphology of spindles and chromosomes. Independent experiments were repeated five times to verify the reliability of results (*n* = 90–100 COCs).

### 2.17 Co‐Staining of Lysosomal and Mitochondrial Distribution

Samples from the oocytes were collected and then followed by three washes with 0.1% PVA‐PBS. Lysosomal dye was mixed with MitoTracker Red CMXRos (a red fluorescent probe for mitochondria), and the oocytes underwent 30 min incubation in an incubator. Subsequently, the oocytes were washed 3 times with 0.1% PVA‐PBS (5 min per wash), then transferred to slides containing Hoechst 33342 anti‐fluorescence quencher and mounted with coverslips coated with lanolin at the four corners. Images were immediately captured under a confocal microscope to observe the co‐staining of oocytes in each group. To guarantee the robustness of the experimental outcomes, we carried out five separate replicates (*n* = 90–100 COCs).

### 2.18 Western Blot

Proteins were extracted using RIPA lysis buffer (Cat#: P0013C, Beyotime Biotechnology, China) and subsequently mixed with SDS‐PAGE loading buffer (Cat#: P0015L, Beyotime Biotechnology, China). The extracted total proteins were subjected to boiling for 5 min to achieve protein denaturation. To begin with, proteins were electrophoresed in the stacking gel for 30 min at a constant 80 V, and subsequently run through a 12.5% separating gel at a constant voltage of 120 V for 90 min. Nextly, the proteins underwent transfer to PVDF membranes (Millipore, ISEQ00010, USA) with a constant current setting of 200 mA over a 150 min duration. Following overnight blocking with 5% BSA in TBST solution, the membranes underwent incubation with primary antibodies, including LC3 (1:1000), P62 (1:1000), and EGR1 (1:500) antibodies. The membranes were rinsed prior to 90 min incubation with HRP‐linked goat anti‐rabbit secondary antibody (Beyotime, China). In the end, chemiluminescence‐based detection of protein signals was performed, with GAPDH (glyceraldehyde 3‐phosphate dehydrogenase) used as the internal reference (housekeeping protein), and the data were processed using AlphaView SA software. The reliability of the findings was validated by performing three independent replicate experiments (*n* = 1000–1200 COCs).

### 2.19 Micro Transcriptome Sequencing

Fresh, Aged, and UDCA samples were collected and subjected to microtranscriptome sequencing (Three biological replicates were set for each group, and 50 oocytes were used in each replicate). Sequencing was performed by Novogene Co. Ltd. (Beijing, China). Three biological replicates were set for each group to ensure the reliability of sequencing results. Raw sequencing data were first subjected to quality control using FastQC, adapter removal, and filtering of low‐quality reads. The clean reads were then mapped to the reference genome using the STAR software, and gene expression quantification was performed with featureCounts. Differential expression analysis between groups was conducted using the DESeq2 package. Differentially expressed genes (DEGs) were screened with the thresholds of *p*_adj_ < 0.05 and |log_2_FoldChange| > 1, followed by GO functional enrichment and KEGG pathway analysis.

### 2.20 Statistical Analysis

In order to ensure result representativeness, at least five parallel replicates were performed for each sample group, unless otherwise specified. All statistical results were analyzed and visualized using GraphPad Prism software. One‐way ANOVA for difference analysis, with statistical significance thresholds designated as *p < 0.05, **p < 0.01, ***p < 0.001, and ns denotes no significant difference. The total sample size and biological replicates for each assay are described in detail in the figure legends.

## 3 Results

### 3.1 UDCA Improved the Quality of Aged Porcine Oocytes

**FIGURE 1:**
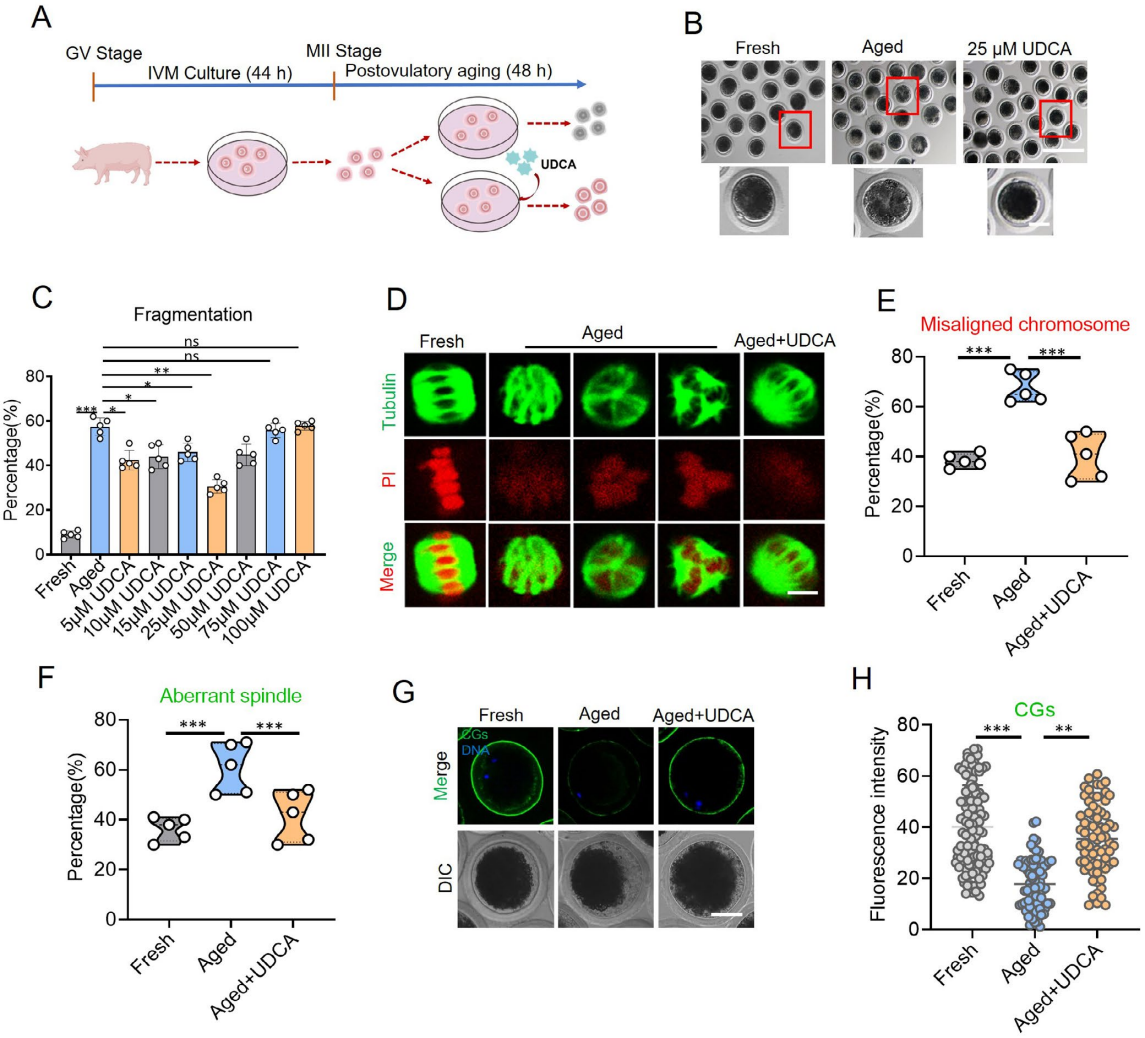
UDCA improved the quality of aged porcine oocytes. Oocytes were incubated in IVM for 44 h, and this group was designated as the Fresh group. Oocytes were cultured in IVM for an extended 48 h under conditions with or without UDCA supplementation. (A) Schematic diagram of in vitro culture and postovulatory aging treatment for porcine oocytes. (B) Typical morphological images of oocytes from Fresh, Aged and Aged +25 μM UDCA groups. Scale bar = 100 μm; Scale bar = 50 μm. (C) The incidence of oocyte fragmentation was quantified in the Fresh (*n* = 260), Aged (*n* = 249), 5 μM UDCA (*n* = 254), 10 μM UDCA (*n* = 234), 15 μM UDCA (*n* = 225), 25 μM UDCA (*n* = 312), 50 μM UDCA (*n* = 232), 75 μM UDCA (*n* = 118) and 100 μM UDCA (*n* = 125) groups. (D) Characteristic imaging results of spindle structure and chromosome alignment in the Fresh, Aged and Aged + UDCA experimental groups. Oocytes were subjected to immunostaining with α‐tubulin‐FITC antibody for spindle visualization, followed by counterstaining with PI to label chromosomes. Scale bars = 75 μm. (E) Chromosome misalignment rates were calculated in Fresh (*n* = 114), Aged (*n* = 123) and UDCA‐treated aged groups (*n* = 116). (F) The proportion of morphologically aberrant spindles was analyzed in the Fresh (*n* = 120), Aged (*n* = 118) and Aged + UDCA (*n* = 123) groups. (G) Characteristic images of CGs distribution patterns in Fresh, Aged and Aged + UDCA groups. Oocytes were stained with FITC‐PNA (green, CGs) and Hoechst 33342 (blue, DNA) Scale bar = 75 μm. (H) The fluorescence intensity of CG signals was measured in Fresh (*n* = 100), Aged (*n* = 104) and Aged + UDCA (*n* = 110) oocytes. Data were subjected to One‐way ANOVA for analysis, and statistically significant results were indicated with asterisks (*p* < 0.05 represented by *, *p* < 0.01 represented by **, *p* < 0.001 represented by ***, and ns denotes no significant difference). Data corresponding to panels C, E, F and H are expressed as mean percentages or values (mean ± SEM) derived from no fewer than five independent experiments. *n* represents the total sample size of biological replicates.

To explore the role of UDCA in aged porcine oocytes, IVM medium was supplemented with UDCA at distinct concentrations (5, 10, 15, 25, 50, 75, and 100 μM) during the oocyte aging period. Oocyte morphology (fragment rate) was analyzed. As shown in Figure 1, fresh oocytes exhibited regular morphology, and many oocytes had the first polar body extruded, with the fragmentation rate of oocytes being around 10.20% (10.20% ± 1.27%). By contrast, the fragmentation rate of aged oocytes was significantly increased, which could be alleviated by supplementation of UDCA (5, 10, 15, 25, 50, 75 and 100 μM). Particularly, the 25 μM UDCA was the most effective in decreasing the aged oocytes‐increased fragmentation rate (35.40% ± 1.87% vs. 62.12% ± 1.59%, *p* < 0.01, Figure 1B,C). Hence, the 25 μM UDCA was applied in subsequent experiments. Nuclear and cytoplasmic maturation are pivotal processes governing oocyte quality, with intimate links to subsequent fertilization success and embryonic developmental competence. Spindle organization, chromosomal alignment, and CGs are key parameters reflecting nuclear and cytoplasmic maturation in oocytes, which were further evaluated in subsequent experiments. As shown in Figure 1, compared with the Fresh group, the abnormal distribution rate of spindle in aged oocytes was significantly increased (35.40% ± 2.73% vs. 60.00% ± 2.98%, *p* < 0.001), while UDCA alleviated this condition (43.40% ± 2.98%, *p* < 0.01, Figure 1D,F). Similarly, some chromosome abnormalities in aged oocytes were restored following UDCA supplementation (67.40% ± 1.78% vs. 42.20% ± 2.35%, *p* < 0.001, Figure 1D,E). A significant decrease in CGs fluorescence intensity was detected by LCA‐FITC staining in aged oocytes, which was partially reversed by UDCA treatment (30.45% ± 2.33% vs. 40.45% ± 1.53%, *p* < 0.01, Figure 1G,H). In summary, these results support that UDCA administration ameliorates the quality of post‐ovulatory aged porcine oocytes.

### 3.2 UDCA Enhanced Mitochondrial Function in Aged Porcine Oocytes

**FIGURE 2:**
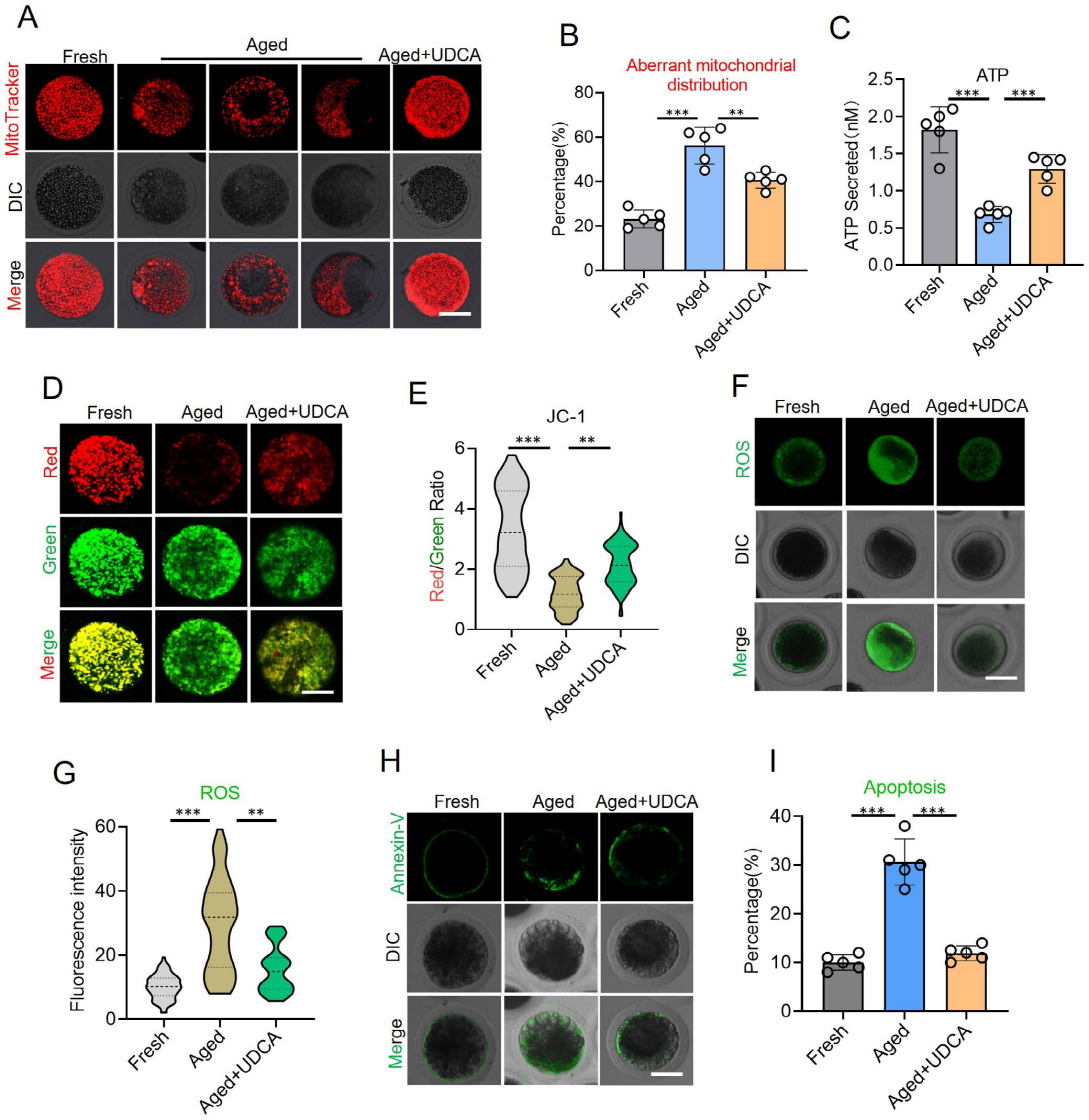
UDCA repaired mitochondrial dysfunction in aged porcine oocytes. (A) Shown are representative images depicting mitochondrial distribution across the Fresh, Aged, and Aged + UDCA groups. Scale bar = 75 μm. (B) The frequency of aberrant mitochondrial distribution was quantified in the Fresh (*n* = 112), Aged (*n* = 120), and Aged + UDCA (*n* = 115) groups. (C) ATP content was determined in Fresh (*n* = 250), Aged (*n* = 261) and Aged + UDCA (*n* = 252) groups. (D) Representative micrographs of Mitochondrial membrane potential in Fresh, Aged and Aged + UDCA groups. JC‐1 staining of oocytes revealed red fluorescence (high mitochondrial membrane potential) and green fluorescence (low mitochondrial membrane potential). Scale bar = 75 μm. (E) The ratio of red to green fluorescence intensity was quantified in the Fresh (*n* = 114), Aged (*n* = 111) and Aged + UDCA (*n* = 112) groups. (F) Representative DCFH‐DA staining images for ROS content in Fresh, Aged and UDCA intervention groups. Scale bar = 75 μm. (G) ROS signal fluorescence intensity was measured in the Fresh (*n* = 150), Aged (*n* = 125), and Aged + UDCA (*n* = 133) cohorts. (H) Characteristic imaging of the apoptotic status of in Fresh, Aged and Aged + UDCA groups. Scale bar = 75 μm. (I) Apoptosis in oocytes from the Fresh (*n* = 147), Aged (*n* = 131) and Aged + UDCA (*n* = 129) groups were quantified and statistically assessed. Statistical analysis of the experimental data was performed via One‐way ANOVA, and asterisks were used to indicate statistically significant variations (*p* < 0.01 represented by **, and *p* < 0.001 represented by ***). The data in B, C, E, G, and I are reported as mean percentage or mean value ± standard error of the mean (SEM), derived from no fewer than five independent experiments. *n* represents the total sample size of biological replicates.

Impaired mitochondrial function is a major factor underlying the reduced quality of porcine oocytes. Subsequently, the main indicators of mitochondrial function, including mitochondrial distribution, MMP and ATP levels were measured. MitoTracker Red staining revealed that aged oocytes had a significantly greater abnormal mitochondrial distribution ratio than the Fresh group (59.40% ± 1.99% vs. 24.53% ± 1.59%, *p* < 0.001, Figure 2A,B). As expected, UDCA supplementation rescued the abnormal mitochondrial distribution in aged oocytes (36.40% ± 1.36% vs. 59.40% ± 1.99%, *p* < 0.01, Figure 2A,B). Conversely, MMP and ATP levels were significantly decreased in the Aged group when compared with the Fresh group and the Aged + UDCA group (*p* < 0.05, Figure 2C–E). The above results show that UDCA repaired mitochondrial dysfunction in aged porcine oocytes.

Mitochondria regulate the metabolism of ROS and participate in apoptosis. Nextly, apoptosis and ROS levels in oocytes were studied. We found that compared with the Fresh group (19.40% ± 1.21%) and Aged + UDCA group (33.60% ± 2.02%) the levels of ROS in aged oocytes (53.00% ± 2.72%, *p* < 0.05, Figure 2F,G) were significantly increased. Consistent with this, relative to the Aged group, UDCA significantly diminished the oocyte apoptotic rate (31.80% ± 2.15% vs. 16.00% ± 0.71%, *p* < 0.001, Figure 2H,I). These results suggest that UDCA can remove excessive ROS accumulation and reduce apoptotic levels in aged porcine oocytes.

### 3.3 UDCA Restored the Early Embryonic Developmental Competence of Aged Porcine Oocytes

**FIGURE 3:**
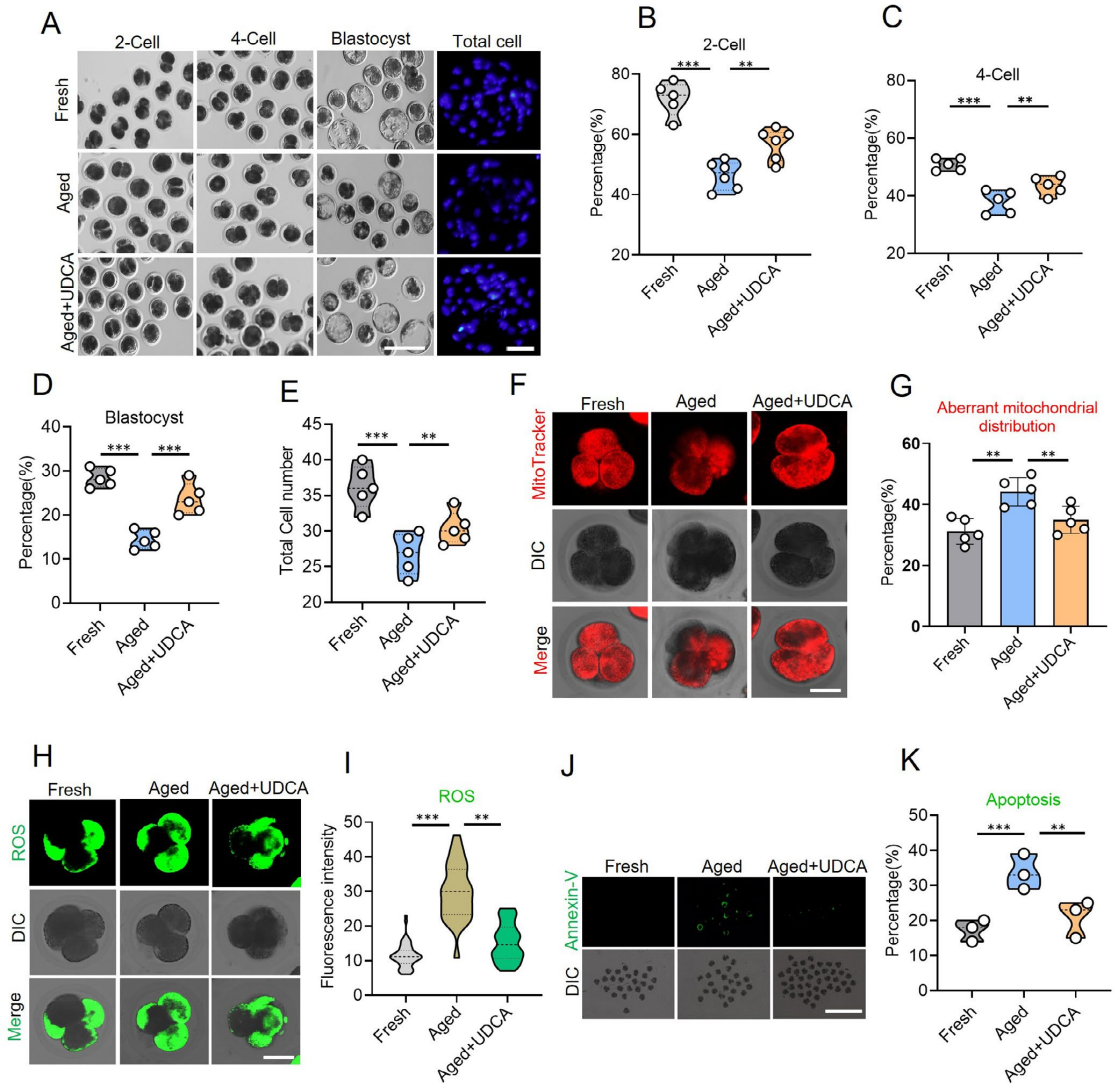
UDCA restored the early embryonic developmental potential of aged porcine oocytes. (A) Characteristic images illustrating different stages of early embryonic development in the Fresh group, Aged group and Aged + UDCA groups. Scale bar = 100 μm; Scale bar = 200 μm. (B) The 2‐cell embryo rate was documented in Fresh (*n* = 150), Aged (*n* = 131) and Aged + UDCA (*n* = 143) groups. (C) The frequency of 4‐cell embryos was documented in Fresh (*n* = 150), Aged (*n* = 131) and Aged + UDCA (*n* = 143) groups. (D) The incidence of blastocyst formation was quantified in Fresh (*n* = 150), Aged (*n* = 131) and Aged + UDCA (*n* = 143) groups. (E) Total cell numbers of blastocysts in Fresh (*n* = 25), Aged (*n* = 24) and UDCA‐treated Aged groups (*n* = 26). (F) Mitochondrial distribution staining showed representative images of mitochondrial function in 4‐cell embryos from Fresh, Aged and Aged + UDCA groups. Scale bar = 75 μm. (G) Statistical analysis was performed on the abnormal distribution rate of mitochondria in the Fresh (*n* = 30), Aged (*n* = 32), and Aged + UDCA (*n* = 35) groups. (H) Typical fluorescence micrographs of ROS in 4‐cell embryos from the Fresh, Aged and Aged + UDCA groups. Scale bar = 75 μm. (I) ROS signal fluorescence intensity in 4‐cell embryos was measured in Fresh (*n* = 31), Aged (*n* = 34) and Aged + UDCA (*n* = 35) groups. (J) Representative images revealed the apoptotic status of 4‐cell embryos in the Fresh, Aged and Aged + UDCA groups. Scale bar = 75 μm. (K) The apoptosis rates of 4‐cell embryos in the Fresh (*n* = 35), Aged (*n* = 32) and Aged + UDCA (*n* = 37) groups were counted. One‐way ANOVA was used for comparative analysis; asterisks denote significant differences. Statistical significance was defined as **p < 0.01, ***p < 0.001. Quantitative data in B, C, D, E, G, I and K were shown as mean ± SEM from a minimum of five independent experiments. *n* represents the total sample size of biological replicates.

Since POA impairs oocyte quality and thereby leads to abnormal embryonic development. Subsequently, the effect of UDCA on the developmental potential of aged oocytes was investigated. Compared with fresh oocytes, aged oocytes showed marked reductions in the rates of 2‐cell, 4‐cell, and blastocyst formation following parthenogenetic activation (*p* < 0.05, Figure 3A–D). However, UDCA supplementation effectively enhanced these developmental rates in the aged oocytes (*p* < 0.05, Figure 3A–D). Similarly, the Aged group exhibited a significantly lower total blastocyst cell number than the Fresh group (23.20% ± 1.07% vs. 40.00% ± 1.51%, *p* < 0.001, Figure 3A,E). As anticipated, UDCA supplementation enhanced the total blastocyst cell number in the Aged group (32.40% ± 1.03% vs. 23.20% ± 1.07%, *p* < 0.01, Figure 3A,E). Meanwhile, UDCA reduced ROS levels and abnormal mitochondrial distribution in 4‐cell embryos (*p* < 0.01, Figure 3F–I). Additionally, early apoptotic levels in 4‐cell embryos were measured via Annexin V staining. Compared with fresh oocytes, aged oocytes exhibited a significantly higher apoptotic rate (9.2% ± 0.86% vs. 24.80% ± 1.60%, *p* < 0.001), which was effectively reduced by UDCA supplementation (16.60% ± 1.44%, *p* < 0.01, Figure 3J,K). These results demonstrate that UDCA can restore subsequent embryonic development in aged oocytes after ovulation.

### 3.4 Microtranscriptomic Analysis Revealed the Regulatory Pathways Underlying the Protective Effect of UDCA on Aged Porcine Oocytes

**FIGURE 4:**
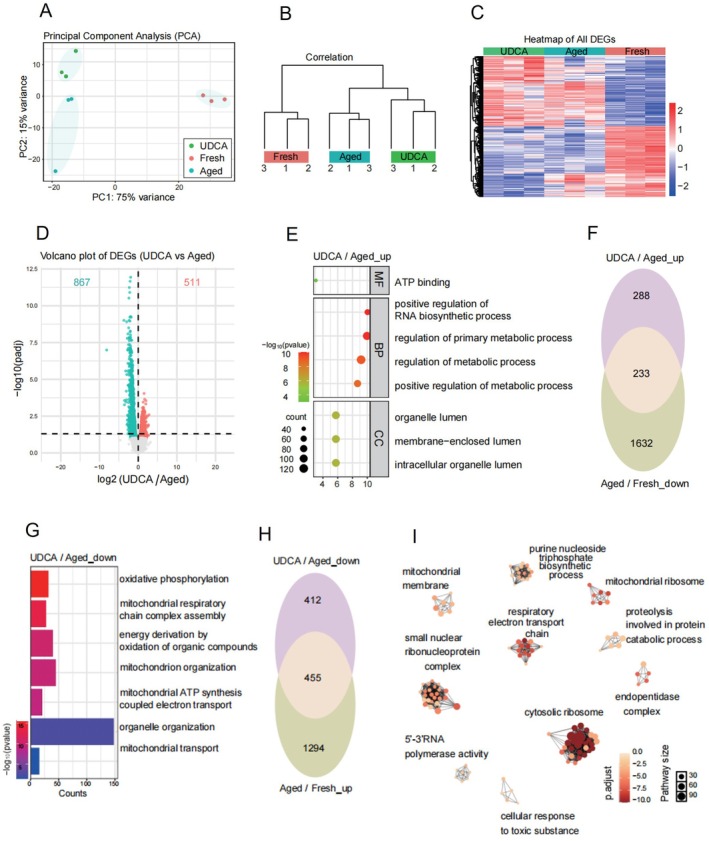
Microtranscriptome analysis revealed the regulatory pathways underlying the effects of UDCA. (A) Principal component (PC) analysis was performed on oocyte‐derived samples categorized into Fresh, Aged, and UDCA (Aged + UDCA) groups. The Fresh, Aged, and UDCA groups are indicated by red, blue, and green circles, respectively. (B) Hierarchical clustering dendrogram showed correlation profiles of Fresh (red), Aged (teal) and UDCA (green) groups. Numbers 1–3 represented subpopulations/replicates, indicating inter and intragroup correlation differences. (C) Heatmap showing the total count of DEGs in porcine oocytes from the Fresh, Aged and UDCA groups. (D) Volcano plot of DEGs between the UDCA group and the Aged group. (E) GO functional enrichment plot of upregulated genes in the UDCA group relative to the Aged group. (F) Venn diagram of upregulated genes in the UDCA group and downregulated genes in the Aged group versus the Fresh group. (G) KEGG pathway enrichment plot of downregulated genes in the UDCA group relative to the Aged group. (H) Venn diagram of downregulated genes in the UDCA group and upregulated genes in the Aged group versus the Fresh group. (I) protein–protein interaction network and functional clustering plot of downregulated genes in the UDCA group relative to the Aged group.

To elucidate the molecular mechanisms responsible for the protective effects of UDCA on aged oocyte quality, we conducted microtranscriptome sequencing analysis of ovulated oocytes from three experimental groups: Fresh, Aged, and UDCA‐treated Aged (Aged + UDCA). PCA analysis showed clear separation of UDCA, Fresh and Aged groups, with tight clustering of biological replicates within each group, indicating significant inter‐group differences and high intra‐group reproducibility (PC1: 75% variance, PC2: 15% variance) (Figure 4A). Hierarchical clustering based on correlation showed distinct grouping of Fresh, Aged, and UDCA samples, with tight clustering of biological replicates within each group, indicating significant inter‐group differences and high intra‐group reproducibility (Figure 4B). Furthermore, Heatmap analysis demonstrated strikingly distinct transcriptomic profiles between Fresh and Aged group oocytes, while the transcriptomic pattern was partially rescued in UDCA‐supplemented oocytes (Figure 4C). Additionally, differential analysis of transcriptomic data between the UDCA group and the Aged group identified 1378 significantly DEGs, among which 867 were significantly downregulated and 511 were significantly upregulated in the UDCA group (Figure 4D). GO enrichment analysis indicated that significantly upregulated genes in the UDCA group were mainly concentrated in biological processes associated with membrane, cell membrane and biomembrane (Figure 4E). Venn diagram analysis of gene set overlap revealed 511 upregulated genes in the UDCA vs. Aged group and 1865 downregulated genes in the Aged vs. Fresh group, with 233 overlapping genes (Figure 4F). In addition, KEGG pathway analysis revealed that downregulated genes in the UDCA group relative to the Aged group were functionally enriched in mitochondrial and energy metabolism‐related pathways (Figure 4G). Venn diagram analysis of their overlap revealed 867 downregulated genes in UDCA vs. Aged and 1749 upregulated genes in Aged vs. Fresh, with 455 overlapping genes (Figure 4H). Protein–protein interaction network and functional clustering analysis of downregulated genes in the UDCA group relative to the Aged group revealed that these genes were mainly enriched in processes such as mitochondrial function regulation, nucleotide metabolism, and RNA metabolism (Figure 4I). Thus, based on the above observations and relevant literature, we put forward the assumption that the improvement of aged oocyte quality by UDCA supplementation may be mediated by autophagy and mitochondrial function.

### 3.5 UDCA Promoted Autophagy in Aged Porcine Oocytes

**FIGURE 5:**
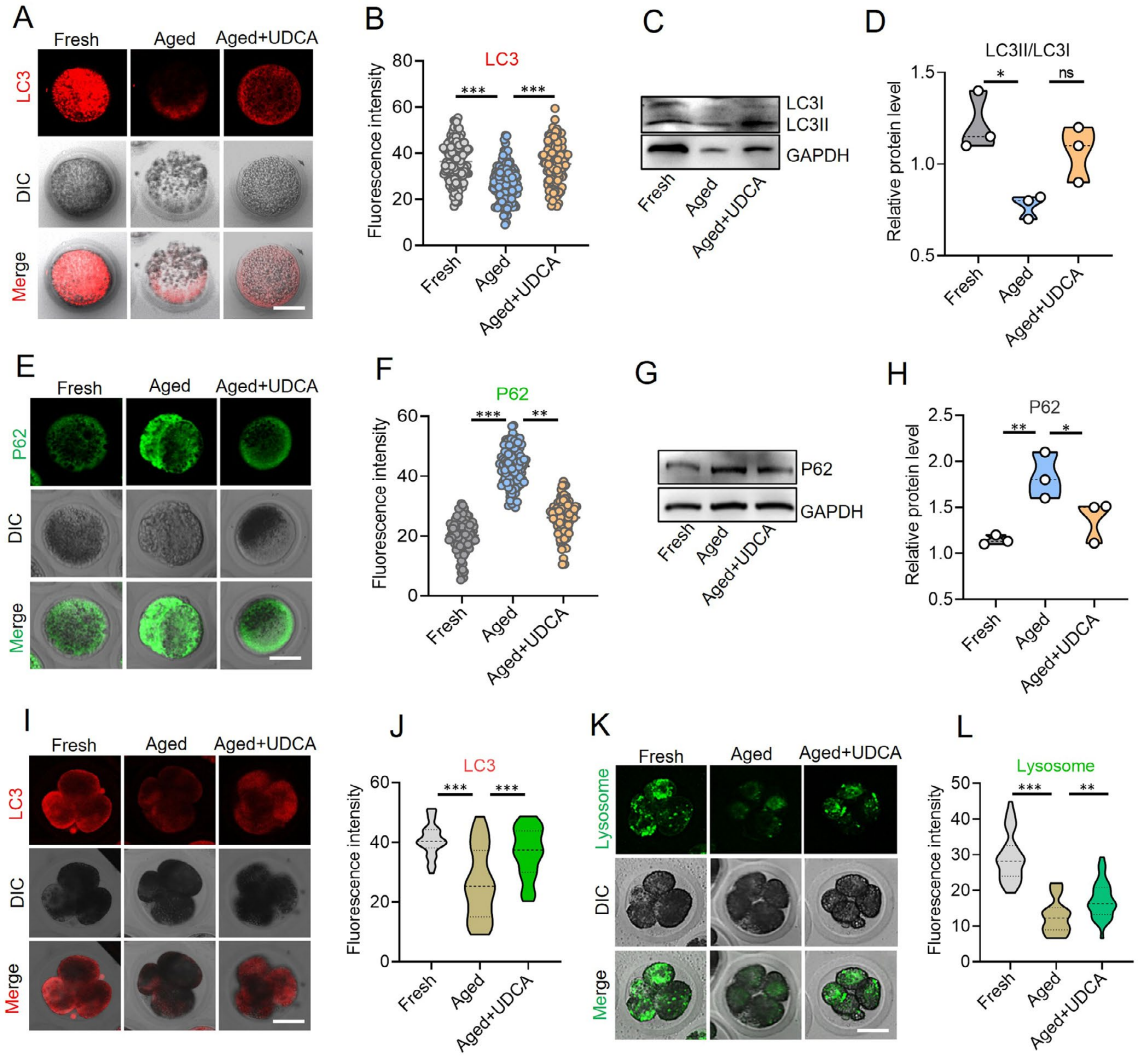
UDCA elevated the autophagy level of aged porcine oocytes. (A) Representative photomicrographs illustrating autophagic activity in Fresh, Aged and Aged + UDCA group oocytes stained with LC3B antibody. Scale bar = 75 μm. (B) The levels of LC3B‐associated fluorescence intensity were determined in oocytes of the Fresh (*n* = 161), Aged (*n* = 151), and Aged + UDCA (*n* = 143) groups. (C) Representative Western blots showing the LC3B expression in oocytes from Fresh, Aged and Aged + UDCA groups. (D) Protein levels of LC3II/I in Fresh, Aged and Aged + UDCA groups. A total of 200 oocytes per group were collected and subjected to immunoblotting for LC3 protein detection. (E) Representative images demonstrated autophagosome staining with an anti‐P62 antibody in oocytes of the Fresh, Aged and Aged + UDCA groups. Scale bar = 75 μm. (F) Quantification of P62 fluorescence intensity was performed in oocytes of the Fresh (*n* = 154), Aged (*n* = 151) and Aged + UDCA (*n* = 153) groups. (G) Representative Western blots showing the P62 expression in oocytes from Fresh, Aged and Aged + UDCA groups. (H) Protein levels of P62 in Fresh, Aged and Aged + UDCA groups. A sample of 200 oocytes per group was collected and analyzed by immunoblotting to quantify P62 protein. (I) Representative immunofluorescence images of 4‐cell embryos from Fresh, Aged and Aged + UDCA groups, demonstrated autophagosome localization with an anti‐LC3 antibody. Scale bar = 75 μm. (J) LC3 signal intensity was measured in 4‐cell embryos from the Fresh (*n* = 32), Aged (*n* = 31) and Aged + UDCA (*n* = 30) groups. (K) Representative images depicted autophagosomes in 4‐cell embryos from the Fresh, Aged and Aged + UDCA groups, stained with LysoTracker Green. Scale bar = 75 μm. (L) Quantitative analysis of lysosomal fluorescent signals in 4‐cell embryos across three groups. Fresh (*n* = 34), Aged (*n* = 31) and Aged + UDCA (*n* = 33) groups. Data were analyzed by One‐way ANOVA. Statistical significance was indicated by asterisks (*p* < 0.05 represented by *, *p* < 0.01 represented by ** *p* < 0.001 represented by ***, and ns denotes no significant difference). Data corresponding to panels B, D, F, H, J and L are expressed as mean percentage or value (mean ± SEM) from no fewer than five independent experiments. *n* represents the total sample size of biological replicates.

To evaluate the influence of UDCA on autophagy in aged oocytes, we quantified the expression of the autophagy markers LC3 and P62 in fresh, aged, and UDCA‐treated aged oocytes. Immunofluorescence and Western blot data demonstrated that LC3B (LC3II/LC3I) protein expression was significantly reduced in aged oocytes, which could be partially restored by UDCA treatment (*p* < 0.05, Figure 5A–D). Conversely, the Aged group exhibited a significant upregulation of P62 protein expression in aged oocytes compared to the Fresh and Aged + UDCA group (*p* < 0.05, Figure 5E–H). Subsequently, we detected the autophagic status of 4‐cell embryos in the Fresh, Aged and Aged + UDCA group. Immunofluorescence staining demonstrated that LC3 expression was markedly reduced in aged 4‐cell embryos (31.20% ± 1.07%) and this decrease was restored after UDCA treatment (39.80% ± 2.52%, *p* < 0.001, Figure 5I,J). One hallmark of the autophagic process is the sequential formation of autophagosomes and their subsequent fusion with lysosomes, resulting in the generation of autolysosome. Nextly, we evaluated lysosomal signaling in 4‐cell embryos by Lyso‐Tracker Green staining. Results from fluorescence imaging and signal quantification indicated that a high abundance of lysosomal signals, accompanied by multiple large foci, existed in fresh 4‐cell embryos (35.40% ± 2.14%, Figure 5K,L). As a stark contrast, the number and size of lysosomal foci were significantly diminished in the context of aging, and this decrease was rescued by UDCA supplementation (18.80% ± 1.16% vs. 27.80% ± 0.86%, *p* < 0.01, Figure 5K,L). The above research results indicate that UDCA can enhance the autophagy of aging oocytes after ovulation.

### 3.6 UDCA Enhanced Mitophagy in Aged Porcine Oocytes

**FIGURE 6:**
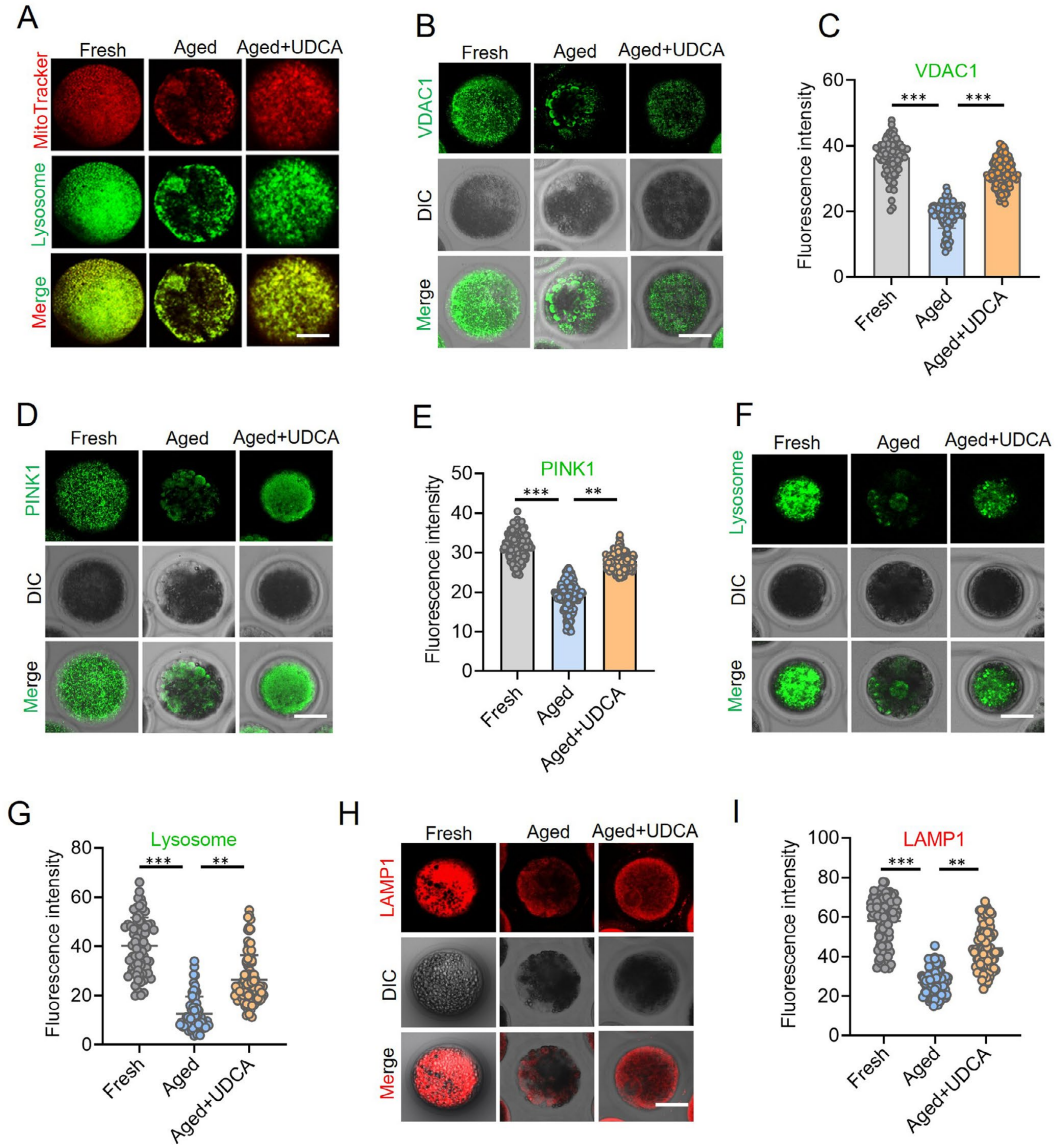
UDCA increased the level of mitophagy in aged porcine oocytes. (A) Representative images revealed mitophagy in oocytes from Fresh, Aged and Aged + UDCA groups, via MitoTracker Red and LysoTracker Green co‐labeling. Scale bar = 75 μm. (B) Representative micrographs of mitophagosomes in oocytes from Fresh, Aged and Aged + UDCA groups, identified via anti‐VDAC1 antibody staining. Scale bar = 75 μm. (C) Separate measurement and calculation of VDAC1 fluorescence intensity were performed for the Fresh (*n* = 154), Aged (*n* = 151), and Aged + UDCA (*n* = 153) groups. (D) Representative immunofluorescence images of oocytes from Fresh, Aged and Aged + UDCA groups were used to show mitophagosomes with an anti‐PINK1 antibody. Scale bar = 75 μm. (E) Fluorescence intensity corresponding to PINK1 was quantified in oocytes of the Fresh (*n* = 154), Aged (*n* = 151) and Aged + UDCA (*n* = 155) groups. (F) Representative images of mitophagosomes stained with LysoTracker Green in oocytes from Fresh, Aged and Aged + UDCA groups. Scale bar = 75 μm. (G) The fluorescence signal intensity from LysoTracker Green was calculated in Fresh (*n* = 124), Aged (*n* = 131) and Aged + UDCA (*n* = 134) groups. (H) Representative images of mitophagosomes stained with anti‐LAMP1antibody from Fresh, Aged and Aged + UDCA groups. Scale bar = 75 μm. (I) Quantitative detection of LAMP1 fluorescence intensity in oocytes among the three experimental groups. Fresh (*n* = 144), Aged (*n* = 141) and Aged + UDCA (*n* = 153) groups. Statistical analysis was performed using one‐way ANOVA, and significant differences are represented by asterisks (**p < 0.01 and ***p < 0.001). Data illustrated in panels C, E, G and I are given as mean percentage or value (mean ± SEM), from a minimum of five independent experimental replicates. *n* represents the total sample size of biological replicates.

Mitophagy constitutes a specific type of autophagy that orchestrates the clearance of dysfunctional mitochondria, in turn maintaining mitochondrial quality. Mitophagy formation in oocytes from each group was evaluated via double staining with MitoTracker Red and Lysotracker Green. We found that oocyte aging impaired the colocalization of mitochondria and lysosomes, which could be restored by UDCA supplementation (Figure 6A). Additionally, fluorescence signals showed that VDAC1 focus signals in fresh oocytes were mainly concentrated in the cytoplasm, while those in aged oocytes were less (45.00% ± 1.92% vs. 26.00% ± 1.76%, *p* < 0.001, Figure 6B,C). On the contrary, UDCA could significantly increase the VDAC1 signal in aged oocytes (48.20% ± 2.75% vs. 26.00% ± 1.76%, *p* < 0.001, Figure 6B,C). Similarly, we observed that the majority of PINK1 focal signals were present in the cytoplasm of fresh oocytes, whereas only a small amount of signal was detected in aged oocytes (40.00% ± 1.70% vs. 19.20% ± 1.69%, *p* < 0.001, Figure 6D,E). As expected, UDCA supplementation effectively enhanced PINK1 signals in aged oocytes (30.00% ± 1.61% vs. 19.2% ± 1.69%, *p* < 0.01, Figure 6D,E). Lyso‐Tracker Green staining revealed markedly lower lysosomal signals in the Aged group than in the Aged + UDCA group (29.80% ± 1.73% vs. 37.80% ± 2.52%, *p* < 0.01, Figure 6F,G). In line with this, UDCA enhanced LAMP1 protein expression in aging‐suppressed oocytes (43.40% ± 2.04% vs. 27.40% ± 2.24%, *p* < 0.01, Figure 6H,I). Collectively, the above data indicate that UDCA reinforces mitophagy activity in porcine aged oocytes.

### 3.7 EGR1‐Mediated Mitophagy Is Essential for UDCA‐Alleviated Damage in Aged Porcine Oocytes

**FIGURE 7:**
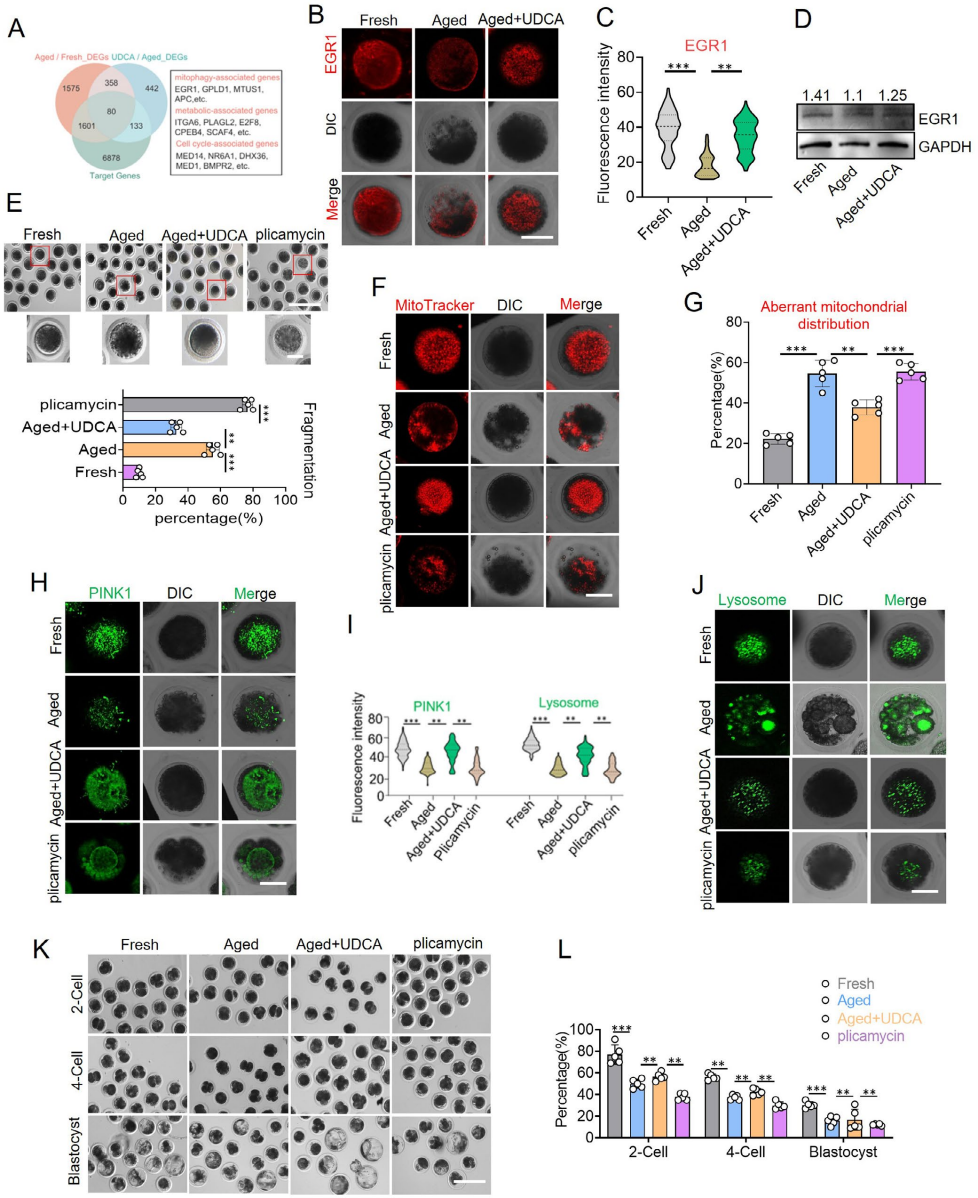
EGR1 upregulation mediated the enhancement of mitophagy by UDCA, which inhibited postovulatory aging in porcine oocytes. (A) Venn diagram of DEGs overlap between Aged/Fresh and UDCA/Aged, with target genes and examples of mitophagy, metabolic and cell cycle‐associated genes. (B) Characteristic visualizations of EGR1 expression levels detected by immunofluorescence staining in Fresh, Aged and Aged + UDCA groups. Scale bar = 75 μm (C) Quantitative analysis was performed on the fluorescence intensity of EGR1 signals in oocytes from the Fresh (*n* = 149), Aged (*n* = 131) and Aged + UDCA (*n* = 133) groups. (D) Representative Western blots showing the EGR1 expression in oocytes from Fresh, Aged and Aged + UDCA groups. (E) Representative images of oocytes from the Fresh, Aged, Aged + UDCA and plicamycin (Aged + UDCA + plicamycin) groups. Scale bar = 100 μm; Scale bar = 50 μm. The fragmentation rate was recorded in the Fresh (*n* = 130), Aged (*n* = 125), Aged + UDCA (*n* = 140) and plicamycin (*n* = 145) groups. (F) Characteristic imaging results showing mitochondrial localization patterns in Fresh, Aged, Aged + UDCA and plicamycin groups. Mito Tracker Red was applied to oocytes as a fluorescent probe to label mitochondria. Scale bar = 75 μm. (G) The incidence of irregular mitochondrial distribution was statistically evaluated in oocytes of fresh, aged, UDCA‐intervened aged and plicamycin treatment groups. Fresh (*n* = 100), Aged (*n* = 97), Aged + UDCA (*n* = 95) and plicamycin (*n* = 102) groups. (H) Representative imaging data of mitophagosomes in oocytes from Fresh, Aged, Aged + UDCA and MMA groups, visualized by anti‐PINK1 antibody immunolabeling. Scale bar = 75 μm. (I) Fluorescence intensity values corresponding to PINK1 and Lysosomal signals were quantified in oocytes from the Fresh (*n* = 144), Aged (*n* = 151), Aged + UDCA (*n* = 154) and plicamycin (*n* = 143) groups. (J) Characteristic imaging results of mitophagosomes identified via staining with LysoTracker Green in oocytes from Fresh, Aged, Aged + UDCA and plicamycin groups. Scale bar = 75 μm. (K) Representative images of different stages of early embryonic development in the Fresh, Aged, Aged + UDCA and plicamycin groups. Scale bar = 100 μm. (L) The rate of 2‐Cell, 4‐Cell embryos and Blastocyst were recorded in Fresh (*n* = 125), Aged (*n* = 131), Aged + UDCA (*n* = 143) and plicamycin (*n* = 133) groups. One‐way ANOVA was employed for data analysis, with asterisks indicating statistically significant outcomes (*p* < 0.01 represented by **, and *p* < 0.001 represented by ***). Data from panels C, E, G, I, and L are represented as mean percentage or value (mean ± SEM), with experiments independently repeated a minimum of five times. *n* represents the total sample size of biological replicates.

Mechanistically, we further explored the specific mechanism by which UDCA enhances mitophagy in aged oocytes. Transcriptomic data showed that EGR1 was significantly downregulated in the Aged group compared with the Fresh group and the UDCA group (Figure 7A, Table S1 and Figure S1). Subsequently, we verified whether UDCA promotes mitophagy by upregulating EGR1, thereby preventing postovulatory aging of porcine oocytes. The protein expression level of EGR1 in aged oocytes was found to be increased by UDCA supplementation, as confirmed by immunofluorescence and Western blot analyses (*p* < 0.05, Figure 7B–D). To investigate whether UDCA promotes the development of aged oocytes via EGR1, porcine oocytes were treated with the EGR1 inhibitor plicamycin. As expected, the beneficial effect of UDCA in rescuing the fragmentation rate of aged oocytes was blocked by plicamycin (32.80% ± 1.65% vs. 57.60% ± 2.50%, *p* < 0.001, Figure 7E). Consistently, the addition of plicamycin reduced the beneficial effects of UDCA on the 2‐cell embryo rate, 4‐cell embryo rate and blastocyst rate (*p* < 0.01, Figure 7K,L). Additionally, UDCA significantly decreased the abnormal distribution of mitochondria in aged oocytes (43.40% ± 1.45% vs. 57.40% ± 1.08%, *p* < 0.01), which was abolished by plicamycin (60.80% ± 1.76%, *p* < 0.01, Figure 7F,G). Furthermore, we observed that UDCA increased the signal intensities of lysosomes and PINK1 (*p* < 0.01, Figure 7H–J). However, the effect of UDCA in promoting mitophagy‐related factors (lysosomes and PINK1) was prevented by plicamycin (*p* < 0.001, Figure 7H–J). Collectively, these data suggest that upregulating EGR1 to promote mitophagy is an important mechanism by which UDCA repairs aged oocyte development.

## 4 Discussion

POA severely impairs oocyte fertilization capacity, compromises cytoplasmic reprogramming in somatic cell nuclear transfer, and ultimately reduces developmental competence of embryos, while also contributing to female fertility decline (Lord and Aitken 2013; Yang et al. 2018). Therefore, elucidating the molecular mechanism of POA and establishing effective intervention strategies are of great significance for enhancing the developmental potential of aging oocytes. This study provides the first direct evidence that UDCA ameliorates the quality of porcine postovulatory aged oocytes by promoting mitophagy through upregulation of EGR1. For the establishment of a reliable aging model, the in vitro aging process was simulated by culturing porcine oocytes for an extended period of 24 or 48 h after the standard 44 h IVM. Previous studies have shown that cytoplasmic fragmentation occurs commonly in aged oocytes, which was one key indicator of the oocyte quality (Zhang et al. 2019). Data generated from our experiments showed that oocyte fragmentation rate was 10.20% (10.20% ± 1.27%) in the Fresh group, increasing to 35% after 24 h and further to 60% after 48 h aging. We found that the proportion of fragmented oocytes slightly increased in the 24 h aged group relative to the fresh control group, with no statistical significance. However, the fragmentation rate of oocytes after 48 h aging showed a markedly greater level than that observed in the fresh group and the difference was remarkable. Correspondingly, in the research on spermidine's improvement of aged porcine oocyte quality, the aging model was constructed via 48 h extended culture post‐maturation, and the oocyte fragmentation rate was 58% (Bai et al. 2024). Hence, combined with our experimental results and previous reports, we ultimately selected extending in vitro culture for 48 h after porcine oocyte maturation as the aging model to simulate the POA process.

This study demonstrated that UDCA not only reduces POA oocyte fragmentation rate and restores normal spindle/chromosome alignment, but also enhances cortical granule fluorescence intensity, ultimately elevating 2‐, 4‐cell and blastocyst rates. Corresponding studies indicate that nicotinamide mononucleotide enhanced aged oocyte quality through the restoration of spindle organization and suppression of chromosomal alignment errors (Miao et al. 2020); furthermore, spermidine treatment enhanced CGs in aged porcine oocytes, thus restoring the developmental capacity of oocytes (Bai et al. 2024). In aged mice, quercetin markedly showed a notable elevation in the 2‐cell rate of oocytes (42.20% ± 2.35% vs. 62.40% ± 1.78%, *p* < 0.001) (Cao et al. 2020). Furthermore, melatonin treatment remarkably enhanced the 4‐cell rate in aged mouse oocytes (38.20% ± 2.05% vs. 54.40% ± 1.28%, *p* < 0.05). In aged porcine oocytes, mangiferin increased the blastocyst rate of aged oocytes (18.40% ± 1.53% vs. 25.20% ± 1.58%, *p* < 0.05) (Yuan et al. 2025). Similarly, apigenin enhanced the inner cell mass cell count in aged oocyte‐derived blastocysts (40.40% ± 2.45% vs. 56.20% ± 2.35%, *p* < 0.001) (Yao et al. 2024). Notably, the uniqueness of this study lies in the fact that, unlike spermidine (Bai et al. 2024) and other conventional interventions for oocyte aging, we are the first to demonstrate the protective effect of the clinically safe drug UDCA on porcine POA oocytes. Furthermore, our subsequent mechanistic investigation moves beyond merely describing phenotypic improvements by identifying the specific molecular target through which UDCA acts.

Mitochondrial dysfunction is the most prominent indicator of impairment in aged oocytes, and this dysfunction is specifically characterized by a significant decrease in MMP, disruption of normal mitochondrial distribution and ATP production capacity (Han et al. 2025; Kobayashi et al. 2023; Miao et al. 2020). Studies have shown that oocyte aging induced abnormal mitochondrial distribution, accompanied by cytoplasmic aggregation and loss of homogeneity, ultimately impaired porcine oocyte quality (Jia et al. 2020). Additionally, aged porcine oocytes exhibited diminished developmental quality along with significantly reduced MMP and ATP levels (Lee et al. 2016). Intriguingly, our study found that UDCA treatment rescued mitochondrial dysfunction in aged oocytes by increasing the normal mitochondrial distribution rate, MMP and ATP levels. Mitochondrial dysfunction triggers apoptosis through disrupting energy homeostasis and cytochrome c release, while concurrently generating excessive ROS due to impaired electron transport chain function (Iba et al. 2025; Meng et al. 2025). Our study demonstrated that porcine oocyte aging promoted a significant increase in both apoptosis and oxidative stress. Interestingly, UDCA treatment significantly reduced both ROS levels and the apoptotic rate in aged porcine oocytes. Relevant literature also confirmed that UDCA inhibited the generation ROS as well as the occurrence of apoptosis (Huang 2021). For example, UDCA reduced the number of apoptotic cells in human colon cancer cells by suppressing the EGFR/Raf‐1/ERK signaling pathway (Im and Martinez 2004). Additionally, UDCA treatment activated the Nrf2 antioxidant signaling pathway, thereby reducing ROS production in vascular smooth muscle cells (Liu et al. 2016). Collectively, this study is the first to systematically verify in germ cells that UDCA combats oocyte aging through a coherent pathway of “repairing mitochondrial function, reducing oxidative damage, and inhibiting apoptosis,” providing a potential strategy for improving the quality of aging oocytes in assisted reproductive technologies.

RNA‐seq analysis suggested that UDCA may affect autophagy by regulating membrane‐related processes. Consistently, we found that UDCA significantly upregulated the expression of autophagy‐related markers LC3B (LC3B‐II/LC3B‐I) and LAMP1, while downregulating P62 expression, thereby elevating autophagic activity in aged porcine oocytes. Previous studies have demonstrated that UDCA can modulate autophagy (Wang et al. 2017). In liver cancer research, UDCA had also been shown to inhibit tumor growth by promoting LC3B expression (Wang et al. 2017). In the field of tumor immunology, UDCA treatment enhanced anti‐tumor immune responses by promoting the P62‐dependent selective autophagy of TGF‐β, a key immunosuppressive factor, thereby inhibiting the differentiation and activation of regulatory T cells (Shen, Lu, et al. 2022; Shen, Wu, et al. 2022). This study reveals a novel mechanism through which UDCA improves the quality of aging oocytes in germ cells by enhancing autophagy levels, which differs from previous findings in somatic cell studies.

Building on this, we further focused on mitophagy. Mitophagy, a specific form of autophagy, refers to the process by which cells selectively recognize, encapsulate and degrade dysfunctional through autophagic mechanisms (D'Arcy 2024). PINK1 and VDAC1 together form the core recognition‐labeling pathway in the initiation stage of mitophagy, ensuring that cells only eliminate dysfunctional mitochondria and maintain mitochondrial quality homeostasis (Yang et al. 2021). Previous studies demonstrated that mitophagy exerted a critical function in reproductive and neurological aging (Markaki et al. 2021). In models of human Alzheimer's disease, downregulation of PINK1 reduced mitophagy, thereby exacerbating neuronal death (Song et al. 2021). In oocytes of aged mice, decreased expression of VDAC1 and reduced number of lysosomes led to reduced mitophagy, ultimately impairing oocyte quality. Correspondingly, our study demonstrated decreased expression of PINK1, VDAC1 and lysosomes in aged porcine oocytes. Notably, this study is the first to reveal that the transcription factor EGR1 serves as a critical mediator for UDCA to promote mitophagy, thereby improving the quality of aged porcine oocytes. Relevant studies demonstrated that Exosi‐EGR1 ameliorated abnormal cardiac structure and function by promoting mitophagy in mouse models of cardiac disease (Huang et al. 2025). Additionally, EGR1 enhanced the radioresistance of hepatocellular carcinoma cells by upregulating its target gene Atg4B to promote autophagy (Peng et al. 2017). Furthermore, transcriptome analysis revealed that the expression level of EGR1 was significantly upregulated in the UDCA group relative to the aging group. Based on the transcriptomic data, we hypothesized that UDCA inhibits the POA of porcine oocytes by upregulating EGR1 to promote mitophagy. To verify this regulatory mechanism, we treated porcine oocytes with the EGR1 specific inhibitor plicamycin, so as to explore whether UDCA alleviates oocyte aging via mediating EGR1. We firstly screened and determined the optimal concentration of plicamycin. Based on published studies, 50, 100, and 200 nM plicamycin have been confirmed to activate apoptosis‐related signaling pathways in HEp‐2 cervical cancer cells, induce cellular apoptosis, and suppress tumor growth (Shen, Lu, et al. 2022, Shen, Wu, et al. 2022). In addition, 200 nM plicamycin has been applied to KKU‐213 cholangiocarcinoma cells to suppress cell growth and eliminate tumor cells via the inhibition of anti‐apoptotic proteins (Choi et al. 2014). Furthermore, in BFTC‐905 and 5637 human urothelial carcinoma cells, 100 nM plicamycin attenuated cell proliferation by downregulating GSTM2 protein expression (Saranaruk et al. 2020) Collectively, plicamycin at 0–200 nM exerts distinct biological effects in various cell models. Within this range, plicamycin efficiently modulates downstream target genes with negligible cytotoxicity. On this basis, we further performed a concentration gradient validation in the porcine oocyte model, with plicamycin treatment groups set at 0, 25, 50, 100, and 200 nM (Figure S2). Subsequently, Following treatment with the EGR1 inhibitor plicamycin in porcine oocytes. As expected, the results showed that inhibition of EGR1 markedly reduced the mitophagy level in UDCA‐treated porcine oocytes. This study presents a key innovation by revealing EGR1 as a novel inducer of mitophagy in aging oocytes and defining the previously unknown “UDCA–EGR1–mitophagy” regulatory axis, which constitutes the central finding of this research.

Current functional investigations of EGR1 are largely restricted to tumor biology, neuroscience, and immunology, whereas its roles and regulatory mechanisms in oocytes remain poorly understood, representing a critical research gap. In reproductive contexts, elevated EGR1 expression in ovarian granulosa cells of aged mice has been shown to trigger the NF‐κB signaling pathway, thereby promoting granulosa cell apoptosis and ultimately accelerating follicular atresia (Yuan et al. 2016). Conversely, in bovine granulosa cells, EGR1 is transcriptionally upregulated in response to FGF stimulation and sustains normal cellular physiology and signaling homeostasis via the ERK1/2 signaling cascade and subsequent regulation of downstream target genes (Han et al. 2017). Collectively, these studies highlight that the biological functions of EGR1 are highly divergent and cell‐type and species‐specific. Nonetheless, its exact roles and regulatory networks in oocytes have not been fully elucidated. Importantly, this study demonstrates for the first time that the transcription factor EGR1 serves as a key mediator through which UDCA regulates mitophagy and thereby improves the quality of aged porcine oocytes.

In this study, we found that UDCA significantly upregulated EGR1 expression in aged oocytes, yet the detailed molecular regulatory mechanism remains to be further elucidated. Based on existing literature, we hypothesize that UDCA may modulate the transcription and translation of EGR1 through multiple synergistic signaling pathways. Previous studies have demonstrated that synthetic UDCA conjugates inhibit cyst formation by suppressing HDAC6 activity and excessive proliferation of cholangiocytes, in both in vivo (rat) and in vitro (human cholangiocyte) models of polycystic liver disease (Caballero‐Camino et al. 2020). Meanwhile, other studies reported that EGR1 maintained normal physiological function in vascular smooth muscle cells under inflammatory conditions by inhibiting HDAC‐mediated H3 deacetylation, increasing H3 acetylation and phosphorylation at its promoter, and establishing a positive feedback loop for self‐transcription (Wang et al. 2010). Based on these findings, we speculate that UDCA may activate EGR1 transcription by inhibiting HDAC activity, reducing histone deacetylation at the EGR1 promoter, and improving chromatin accessibility. In addition to HDAC‐related pathways, the regulatory relationship between UDCA and EGR1 may also involve the Nrf2 signaling pathway. Studies have shown that UDCA markedly activates the Nrf2 pathway, thereby upregulating the expression of various antioxidant, detoxification, and transporter proteins in mouse hepatocytes and exerting hepatoprotective effects (Okada et al. 2008). In breast cancer cells, EGR1 promoted erastin‐induced ferroptosis and exerted antitumor effects by activating the Nrf2‐HMOX1 pathway (Lin et al. 2024). Collectively, these findings suggest that the AMPK/Nrf2 axis is likely another critical molecular mechanism underlying the upregulation of EGR1 transcription by UDCA. Furthermore, the MAPK signaling pathway may also participate in the regulation of EGR1 by UDCA. Evidence has shown that UDCA inhibits EGF‐ or DCA‐induced activation of the EGFR‐MAPK pathway, thereby exerting cytoprotective and chemopreventive effects and suppressing colon tumorigenesis (Centuori and Martinez 2014). Meanwhile, in primary mouse hepatocytes and in vivo cholestasis models, bile acids significantly upregulated EGR1 expression by activating the MAPK/ERK pathway, which was involved in hepatic cholestatic stress and inflammatory responses (Allen et al. 2010). Accordingly, we further hypothesize that UDCA may indirectly upregulate EGR1 expression by modulating the MAPK/ERK signaling pathway. In conclusion, the regulation of EGR1 by UDCA exhibits obvious cell‐type specificity. Therefore, we will focus on exploring the precise molecular mechanism by which UDCA regulates EGR1 in porcine oocytes in the future.

Limitations of this study and future research directions are summarized below: Further explore the specific mechanism by which EGR1 regulates mitophagy. Currently, we have found that EGR1 was capable of delaying the aging of porcine oocytes through promoting mitophagy; however, its downstream target genes and related signaling pathways remain unclear. (1) Future studies should focus on elucidating how EGR1 precisely regulates key autophagy factors through specific transcriptional programs at the transcriptional level, thereby systematically defining its functional pathway during porcine oocyte aging. (2) Verify the practical application potential of UDCA in animal livestock through rigorous in vivo animal experiments. A critical next step is to validate UDCA as a novel reproductive modulator by determining its effects on sow fertility under postovulatory aging, with a focus on conception rate, litter size and offspring health. (3) From the perspective of translational application, in livestock, screen the low‐cost active metabolites of UDCA to reduce the application cost and promote its wide application in animal husbandry. In human assisted reproduction, identifying approved compounds with UDCA‐like functions will provide candidate drugs for novel oocyte quality improvement strategies, thereby collectively advancing reproductive technologies for both humans and livestock.

## 5 Conclusion

In conclusion, we have demonstrated that UDCA can prevent the aging of porcine oocytes. UDCA promoted mitophagy via upregulating EGR1, inhibited mitochondrial dysfunction, ROS, and apoptosis, thus enhancing the quality of aged and impaired oocytes. These data indicated that UDCA‐induced mitophagy promotion via EGR1 upregulation is a potential strategy for preventing reproductive performance impairment caused by aging. Our study provides an important theoretical basis and potential targets for subsequent optimization of in vitro oocyte culture systems, improvement of livestock reproductive efficiency, and exploration of strategies to enhance oocyte quality in assisted reproductive technologies.

## Author Contributions

Y.Z.: drafting the manuscript and conducting the experimental work. Q.H. Undertaking the investigation work involved in the study. Y.L. Carrying out RNAseq data processing and analysis. X.L. Participating in manuscript revision and editing. W.S. and S.C. Compiling the original manuscript and taking charge of project administration. All authors have critically reviewed the manuscript and consented to the final version for submission.

## Funding

This work was supported by the National Key Research and Development Program of China (2023YFD1300504), the Natural Science Foundation of Shandong Province (ZR2025QC270), the Start‐up Fund for High‐level Talents of Qingdao Agricultural University (1124002), and Shandong Modern Agricultural Industry Technology System—Swine Innovation Team (SDAIT‐08‐02) of China.

## Conflicts of Interest

The authors declare no conflicts of interest.

## Supporting information


**Figure S1:** Statistical significance analysis plot of EGR1 TPM values in the Fresh, Aged, and UDCA groups. (A) The mRNA expression of EGR1 was detected by transcriptome analysis in fresh, aged, and UDCA‐treated aged porcine oocytes. TPM values were used to represent gene expression levels. One‐way ANOVA was employed for data analysis, with asterisks indicating statistically significant outcomes (*p* < 0.01 represented by **). Data from panels A is represented as mean percentage or value (mean ± SEM), with experiments independently repeated a minimum of three times.
**Figure S2:** Determination of the optimal concentration plicamycin. (A) Typical morphological images of oocyte from Aged, Aged + UDCA and 50 nM plicamycin groups. Scale bar = 100 μm; Scale bar = 50 μm. (B) The incidence of oocyte fragmentation was quantified in the, Aged (*n* = 249), Aged + UDCA (*n* = 312), 25 nM plicamycin (*n* = 253), 50 nM plicamycin (*n* = 258), 100 nM plicamycin (*n* = 263) and 200 nM plicamycin (*n* = 265) groups. One‐way ANOVA was employed for data analysis, with asterisks indicating statistically significant outcomes (*p* < 0.01 represented by **, *p* < 0.001 represented by ***, and ns represented no significant difference). Data from panels B is represented as mean percentage or value (mean ± SEM), with experiments independently repeated a minimum of five times. *n* represents the total sample size of biological replicates.

**Table S1:** Statistical table of relative EGR1 expression levels.

## Data Availability

The data that support the findings of this study are available on request from the corresponding author. The data are not publicly available due to privacy or ethical restrictions.
