# Supplementary material for: EGR1 Mediates Ursodeoxycholic Acid‐Promoted Mitophagy to Prevent Postovulatory Aging of Porcine Oocytes
Source: Aging Cell. 2026 Jul 3;25(7):e70612. doi: 10.1111/acel.70612 (PMC13330137; doi:10.1111/acel.70612)
Supplement: Supplementary file 1 — Figure S1: Statistical significance analysis plot of EGR1 TPM values in the Fresh, Aged, and UDCA groups. (A) The mRNA expression of EGR1 was detected by transcriptome analysis in fresh, aged, and UDCA‐treated aged porcine oocytes. TPM values were used to represent gene expression levels. One‐way ANOVA was employed for data analysis, with asterisks indicating statistically significant outcomes (p < 0.01 represented by **). Data from panels A is represented as mean percentage or value (mean ± SEM), with experiments independently repeated a minimum of three times. Figure S2: Determination of the optimal concentration plicamycin. (A) Typical morphological images of oocyte from Aged, Aged + UDCA and 50 nM plicamycin groups. Scale bar = 100 μm; Scale bar = 50 μm. (B) The incidence of oocyte fragmentation was quantified in the, Aged (n = 249), Aged + UDCA (n = 312), 25 nM plicamycin (n = 253), 50 nM plicamycin (n = 258), 100 nM plicamycin (n = 263) and 200 nM plicamycin (n = 265) groups. One‐way ANOVA was employed for data analysis, with asterisks indicating statistically significant outcomes (p < 0.01 represented by **, p < 0.001 represented by ***, and ns represented no significant difference). Data from panels B is represented as mean percentage or value (mean ± SEM), with experiments independently repeated a minimum of five times. n represents the total sample size of biological replicates. [file ACEL-25-e70612-s001.docx]

Table S1


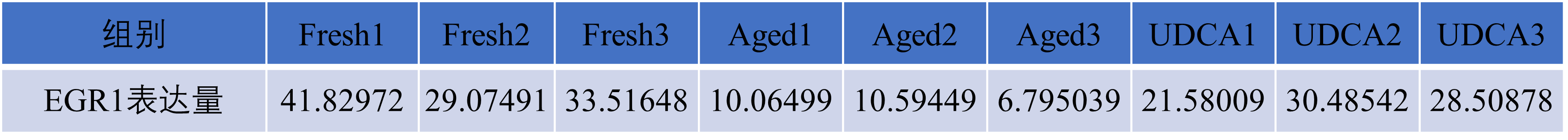
Table S1. Statistical table of relative EGR1 expression levels

Table S1. The expression of EGR1 was determined by transcriptome sequencing in fresh, aged, and UDCA-treated porcine oocytes, with three biological replicates per group.


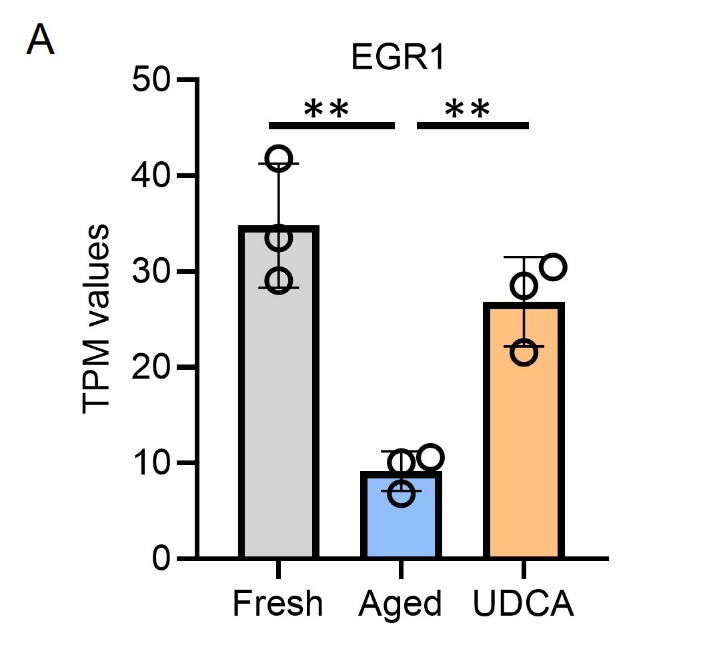
Fig. S1

Fig S1. Statistical significance analysis plot of EGR1 TPM values in the Fresh, Aged, and UDCA groups. A. The mRNA expression of EGR1 was detected by transcriptome analysis in fresh, aged, and UDCA-treated aged porcine oocytes. TPM values were used to represent gene expression levels. One-way ANOVA was employed for data analysis, with asterisks indicating statistically significant outcomes. (*P* < 0.01 represented by **). Data from panels A is represented as mean percentage or value (mean ± SEM), with experiments independently repeated a minimum of three times.

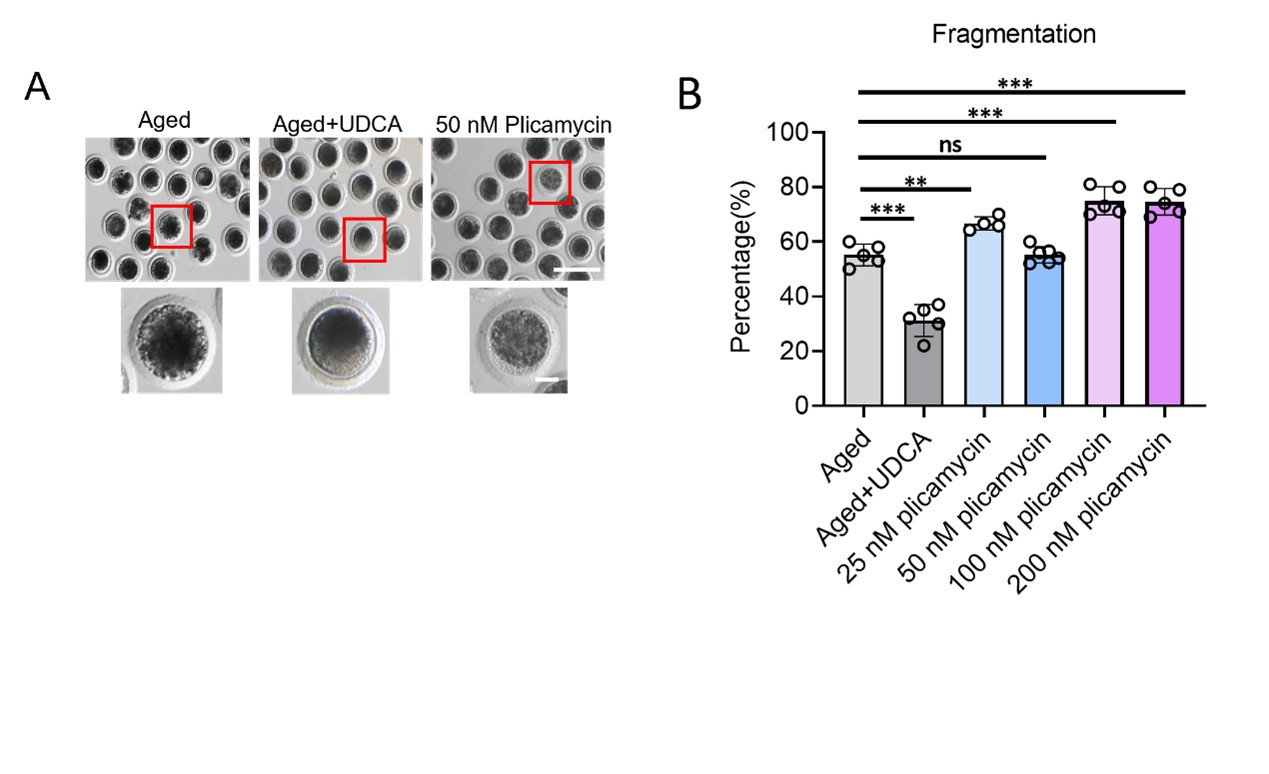
Fig. S2

Fig. S2. Determination of the optimal concentration plicamycin. A. Typical morphological images of oocyte from Aged, Aged+UDCA and 50 nM plicamycin groups. Scale bar = 100 μm; Scale bar = 50 μm. B. The incidence of oocyte fragmentation was quantified in the, Aged (n = 249), Aged+UDCA (n = 312), 25 nM plicamycin (n = 253), 50 nM plicamycin (n = 258), 100 nM plicamycin (n = 263) and 200 nM plicamycin (n = 265) groups. One-way ANOVA was employed for data analysis, with asterisks indicating statistically significant outcomes. (*P* < 0.01 represented by **, *P* < 0.001 represented by ***, and ns represented no significant difference). Data from panels B is represented as mean percentage or value (mean ± SEM), with experiments independently repeated a minimum of five times. n represents the total sample size of biological replicates.
